# A Bacteriophage‐Derived Primase‐Helicase Orchestrates Plant Organellar DNA Replication

**DOI:** 10.1111/ppl.70379

**Published:** 2025-07-07

**Authors:** Carlos M. Morales‐Vázquez, Mayra A. Dagio‐Hernandez, Laura D. Camacho‐Manriquez, Antolin Peralta‐Castro, Claudia D. Raygoza, Diana Solano‐Argüello, Josue D. Mora‐Garduño, Rogelio Gonzalez‐Gonzalez, Humberto Herrera‐Ubaldo, Corina Díaz‐Quezada, Alfredo Cruz‐Ramírez, Stefan de Folter, José Antonio Pedroza‐García, Luis G. Brieba

**Affiliations:** ^1^ Centro de Investigación y de Estudios Avanzados del Instituto Politécnico Nacional Unidad de Genómica Avanzada Irapuato México; ^2^ Departamento de Bioquımica, Facultad de Quımica Universidad Nacional Autonoma de Mexico Ciudad de Mexico Mexico

**Keywords:** hexameric primase‐helicase, plant mitochondrial replisome, protein–protein interaction

## Abstract

The mechanisms underlying the assembly and regulation of enzymatic complexes responsible for plant organellar DNA replication remain poorly characterized. Unlike the monophyletic origin of the gene products involved in animal mitochondrial replication, derived from T‐odd bacteriophages, plant organellar DNA replication relies on genes either unique to plants or with origins traceable to bacteria and bacteriophages. Here, we demonstrate that the bacteriophage‐related primase‐helicase from *Arabidopsis thaliana* (AtTwinkle) is essential for double‐stranded DNA unwinding. AtTwinkle functionally interacts with bacterial‐related organellar DNA polymerases (AtPolIs), which lack the ability to unwind large regions of dsDNA, coupling DNA unwinding to processive DNA synthesis at the leading strand of the replisome. Analysis of two T‐DNA insertion mutants of AtTwinkle reveals distinct phenotypic outcomes; these mutant lines are hereafter referred to as *ph*. The *ph1* (−/−) mutant, which carries a T‐DNA insertion in the 5´ UTR region, is viable and exhibits no noticeable developmental differences compared to wild‐type plants. In contrast, the *ph2* mutant, with a T‐DNA insertion in the 19th exon, displays embryo lethality. Despite these differences, both *ph1* (−/−) and heterozygous *ph2* (+/−) mutants show a reduction in organellar DNA copy numbers under non‐stress conditions and exhibit heightened sensitivity to DNA‐damaging agents. In summary, our findings demonstrate that AtTwinkle is essential for organellar DNA replication. The heightened sensitivity of insertion mutants to organelle‐specific genotoxic agents indicates that loss of AtTwinkle function reduces the availability of template DNA necessary for double‐strand break (DSB) repair. Collectively, our findings reveal that two proteins of distinct evolutionary origins—AtTwinkle and plant organellar DNA polymerases—coevolved to coordinate DNA replication in plant organelles.

## 1 Introduction

DNA replication is orchestrated through the coordinated activities of DNA polymerases, primases, helicases, and single‐stranded DNA‐binding proteins (SSBs) within a macromolecular complex known as the replisome (Zhang, Lee, and Richardson 2011; Yang et al. 2019). The simplest replisomes characterized thus far are those of bacteriophage T7 and human mitochondria (Korhonen et al. 2004; Wanrooij and Falkenberg 2010; Lee and Richardson 2011). Plant mitochondria and chloroplasts originated through endosymbiotic events. The endosymbiosis of an ancestral α‐proteobacterium gave rise to mitochondria in heterotrophic eukaryotic cells, which subsequently evolved into animals and yeast. A subsequent endosymbiotic event involving a cyanobacterium led to the emergence of photosynthetic eukaryotes, including plants (Cermakian et al. 1996; Archibald 2009; Gray 2012). In yeast and animals, mitochondrial DNA replication is facilitated by enzymes evolutionarily related to bacteriophage T7 (Shutt and Gray 2006a). However, unlike this monophyletic origin, the predicted replisome components in plant organelles exhibit diverse evolutionary origins (Brieba 2019).

The minimal replisome in plant organelles is predicted to consist of the organellar DNA polymerases, replicases that are phylogenetically related to bacterial DNA polymerases (DNAP) and not to bacteriophage DNAPs (Moriyama et al. 2011; Czernecki et al. 2023), a primase‐helicase related to T‐odd bacteriophages, single‐stranded DNA‐binding proteins (SSBs), plant‐specific organellar single‐stranded DNA binding proteins (OSB), and plant‐specific Whirly proteins (Krause et al. 2005; Zaegel et al. 2006; Marechal et al. 2009; Lassen et al. 2011; Cupp and Nielsen 2014). Most of these proteins are encoded by the nuclear genome and are imported into both mitochondria and chloroplasts via dual‐targeting sequences, as demonstrated by seminal GFP‐based localization studies (Christensen et al. 2005).

In most eukaryotic organelles, DNA replication requires an ortholog of the bacteriophage T7 DNA primase‐helicase, commonly referred to as Twinkle (T7 gp4‐like protein with an intramitochondrial nucleoid localization). Structural studies have demonstrated that human Twinkle (HsTwinkle) and T7 primase‐helicase assemble into a heterogeneous mixture of oligomeric states, including hexamers, heptamers, and octamers (Sawaya et al. 1999; Toth et al. 2003; Shutt and Gray 2006a; Fernandez‐Millan et al. 2015; Riccio et al. 2022). In HsTwinkle, these distinct conformations are believed to correspond to different stages of helicase loading onto forked DNA substrates (Li et al. 2022; Riccio et al. 2022). According to the “monomer ejection” model, Twinkle predominantly exists as an octamer in solution. Upon encountering a forked DNA structure, one monomer is released, resulting in a heptameric form that adopts an open‐ring conformation. This intermediate is thought to facilitate the ejection of a second monomer, thereby generating a hexamer that is competent for DNA unwinding (Riccio et al. 2022).

**FIGURE 1:**
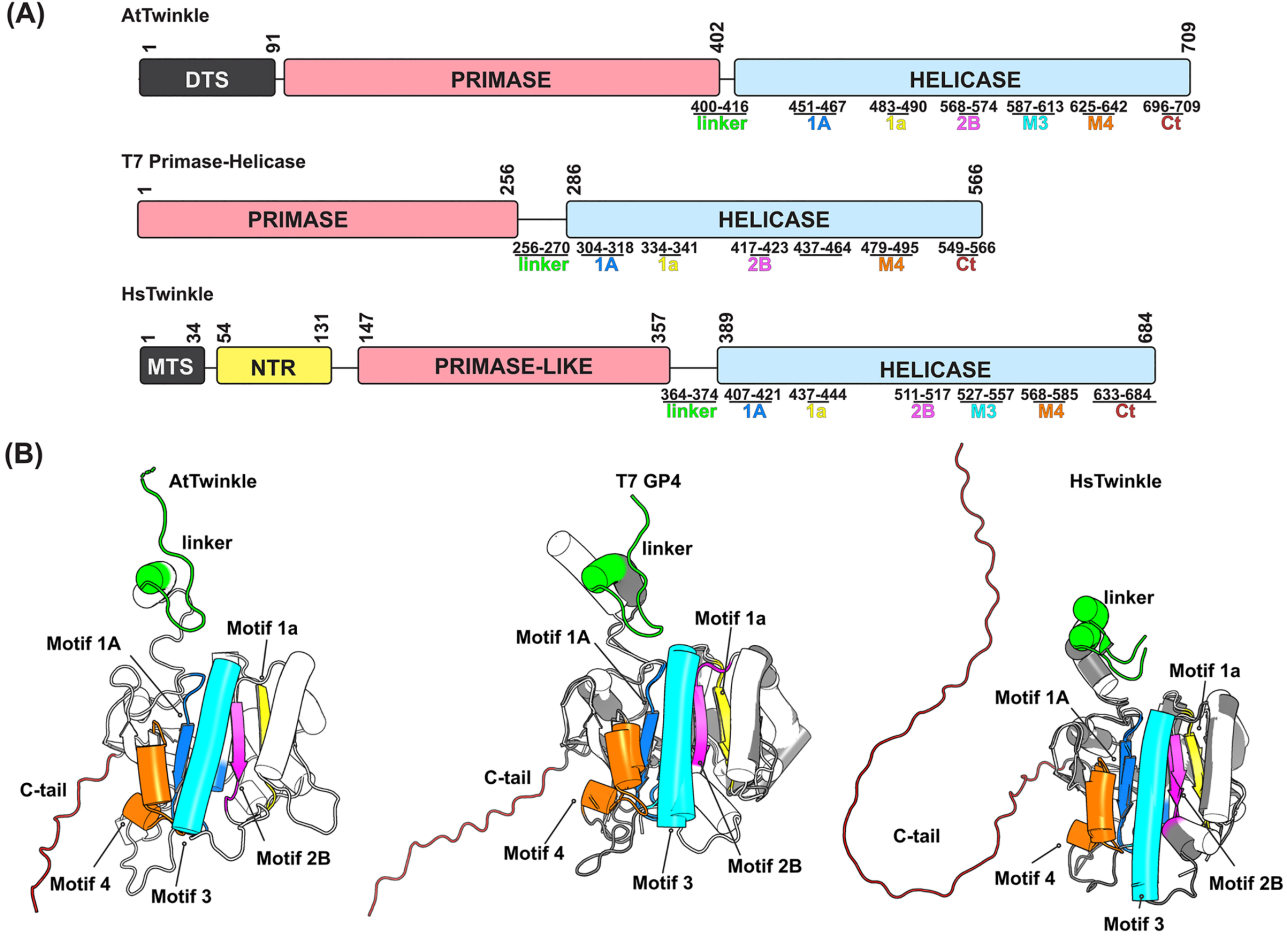
Domain assembly of AtTwinkle in comparison to the T7 Primase‐Helicase and Human (Hs)Twinkle and structural comparison of their helicase domains. (A) Schematic representation of AtTwinkle illustrating its predicted primase and helicase domains, including the dual‐targeting sequence (DTS), in comparison with the structurally characterized domains of bacteriophage T7 primase‐helicase and HsTwinkle. The five conserved motifs in family SF4 of DNA helicases (motif 1A, 1a, 2B, M3, and M4) are indicated. Motifs I and Ia correspond to the Walker A motif, which mediates ATP binding, while motif 2B corresponds to the Walker B motif, essential for ATP hydrolysis, motif M3 is involved in hydrogen bonding and stacking interactions with DNA bases, and motif 4 participates in nucleotide binding. These conserved motifs are shared to varying degrees across all four helicase families and are critical for their enzymatic activity (Gorbalenya et al. 1989; Hall and Matson 1999). In HsTwinkle, mitochondrial localization is directed by a mitochondrial targeting sequence (MTS), and the primase domain has functionally diverged into a non‐catalytic region comprising an N‐terminal region (NTR) and a degenerate primase‐like subdomain. (B) Structural modeling of the helicase domain of AtTwinkle in comparison to HsTwinkle and T7 helicase. The conserved motifs present in T7 primase‐helicase and HsTwinkle are indicated by arabic numerals.

Mutations in HsTwinkle result in mitochondrial DNA depletion and aberrant DNA re‐organization that induces mitochondrial dysfunctions and several neuropathies (Peter and Falkenberg 2020; Zhang et al. 2024). Twinkle from the plant model *Arabidopsis thaliana* (AtTwinkle) is a nuclear‐encoded protein that localizes to both mitochondria and chloroplasts (Christensen et al. 2005). This enzyme exhibits helicase and primase activities, suggesting its involvement in the leading‐ and lagging‐strand DNA synthesis of the mitochondrial and chloroplast genomes (Cao 2012; Diray‐Arce et al. 2013; Towle‐Weicksel et al. 2014; Peralta‐Castro et al. 2017). The primase activity of AtTwinkle and T7 primase‐helicase is mediated by catalytic residues within their RNA polymerase (RNAP) and Zinc Finger (ZFD) subdomains, which are responsible for catalysis and template recognition, respectively (Diray‐Arce et al. 2013; Peralta‐Castro et al. 2017; Peralta‐Castro et al. 2023). In contrast to their plant and bacteriophage counterparts, metazoan Twinkle proteins lack primase activity due to the absence of key catalytic residues in the RNAP subdomain and the loss of metal‐coordinating residues in the ZFD. These structural modifications have led to the evolution of a non‐catalytic N‐terminal region (NTR) and a primase‐like subdomain (Shutt and Gray 2006a; Riccio et al. 2022). Albeit the NTR and primase‐like domain of HsTwinkle are not active, these structural elements are important to promote an oligomeric assembly and to couple helicase‐DNA polymerization in human mitochondria (Figure 1A).

Thus, HsTwinkle, AtTwinkle, and bacteriophage T7 primase‐helicase are modular enzymes comprising distinct primase or primase‐like and helicase domains connected by a flexible amino acid linker (Diray‐Arce et al. 2013; Peralta‐Castro et al. 2017; Figure 1A). The helicase domain of AtTwinkle shares 21% and 23% amino acid sequence identity with the helicase domains of T7 primase‐helicase and HsTwinkle and retains the four conserved motifs characteristic of the superfamily 4 DNA helicases (Hall and Matson 1999; Figures S1 and S2, and Figure 1B).

In bacteriophage T7 and animal mitochondria replisomes, the leading‐strand DNA polymerase (DNAP) interacts with T7 primase‐helicase or Twinkle for DNA unwinding and template delivery into the polymerase active site (Gao et al. 2019). In bacteriophage T7, the interaction between the hexameric T7 DNA primase‐helicase and T7 DNA polymerase is facilitated by a highly acidic C‐terminal tail of the T7 DNA primase‐helicase and two positively charged regions of the T7 DNAP (Hamdan et al. 2005; Zhang, Lee, Zhu, et al. 2011; Gao et al. 2019).

In *Arabidopsis thaliana* , two organellar DNA polymerases, AtPolIA and AtPolIB, which arose from a recent duplication event, are dual targeted to mitochondria and chloroplasts. AtPolIs share over 70% amino acid identity and represent the sole DNA polymerases present in plant organelles. These polymerases are essential for DNA replication and repair in both organelles (Parent et al. 2011). In contrast to animal replicative DNA polymerases, plant organellar DNA polymerases do not share a phylogenetic origin with T‐odd bacteriophage DNA polymerases (Czernecki et al. 2023). AtPolIs are multifunctional enzymes capable of participating in base excision repair, micro‐homology mediated end‐joining, and translesion DNA synthesis (Baruch‐Torres and Brieba 2017; Trasvina‐Arenas et al. 2018; Peralta‐Castro et al. 2020). According to the relevance of replisome assembly in which primase‐helicases and DNA polymerase interactions are indispensable for coordinated DNA replication, we predict a functional interaction between AtTwinkle and AtPolIs. Herein we dissect the functional interactions between the plant organellar DNA polymerases and AtTwinkle and characterize its function in vivo using *twinkle* mutant lines. Our data indicate that AtTwinkle is an indispensable component necessary for replisome assembly in plant organelles, and it is essential to maintain the integrity of organelle DNA and to plant survival.

## 2 Material and Methods

### 2.1 Gene Subcloning

The nucleotide sequence encoding the processed form of AtTwinkle (residues 92–709; AT1G30680) was codon‐optimized for bacterial protein expression and subsequently cloned via specific restriction enzymes into a pET19b vector. Additionally, the nucleotide sequences for the AtHelicase (residues 402–709) were subcloned into a modified pET28‐SUMO vector. Both gene fragments were subcloned between the *Nde* I and *BamH* I restriction sites. The purification of AtTwinkle and AtPolIs proteins was performed according to previously described protocols (Baruch‐Torres and Brieba 2017; Garcia‐Medel et al. 2019; Peralta‐Castro et al. 2020). In both cases, codon optimization enhances the expression of the recombinant protein, while the addition of the SUMO tag improves its solubility. Incubation with a precision protease then removes the SUMO tag, leaving only a few extra amino acids behind.

### 2.2 Structural Modeling

Structural models were obtained from the AlphaFold Protein Structure Database (AFDB) or generated using AlphaFold3 (Jumper et al. 2021; Abramson et al. 2024). In the latter case, structural predictions were performed using protein sequences lacking the organellar targeting signal. The Predicted Local Distance Difference Test (pLDDT) revealed notable differences between AtPolIs and AtTwinkle structural models. pLDDT scores are interpreted as follows: values above 90 indicate very high confidence; scores between 90 and 70 reflect confident predictions; 70–50 indicate low confidence; and scores below 50 suggest poor model reliability. For AtPolIs, a specific segment of residues 30–257 was previously suggested to fold as a disordered region (Baruch‐Torres and Brieba 2017), showed pLDDT values below 50, suggesting low confidence in that region, whereas the remainder of the protein exhibited scores above 70, indicating high confidence folding in the exonuclease and polymerase domains. In contrast, AtTwinkle displayed pLDDT values below 50 for the first six residues after the processing of the targeting sequence (residues 91–118) and the last C‐terminal 10 residues. The Zinc finger subdomain (residues 118–173) showed intermediate confidence with scores ranging from 70 to 50, while the RNAP (residues 174–400) and linker plus helicase domains (401–701) displayed pLDDT values between 90 and 70, indicating confident structural predictions (Figure S2).

### 2.3 Heterologous Protein Expression and Purification

For in vitro experiments, AtTwinkle and AtHelicase protein expression were carried out in an *E. coli* BL21(*DE3*) strain supplemented with pKJ7 chaperones (Takara) to promote proper protein folding. Protein expression was induced once the cell cultures reached an OD_600_ of 0.6 by adding 1 mM IPTG, followed by a 16‐h incubation at 16°C. After incubation, cells were harvested via centrifugation using a Beckman JA‐10 rotor at 4500 *g* for 30 min. The pellet was resuspended in lysis buffer (25 mM sodium phosphate buffer pH 7.5, 500 mM NaCl, 5% glycerol, and 1 mM PMSF). Cells were subjected to three freeze–thaw cycles in the presence of 0.5 mg mL^−1^ lysozyme. The cleared lysate was obtained through centrifugation at 15,000 *g* for 45 min, and the supernatant was filtered using a 0.45 μm PVDF (Hydrophobic Polyvinylidene Fluoride) syringe filter and applied to a 1 mL Ni‐NTA affinity column and washed with 50 mL of lysis buffer supplemented with 25 mM imidazole. Elution was performed in lysis buffer supplemented with 500 mM imidazole. The proteins were diluted to 50 mM NaCl, then loaded onto a HiTrap HP heparin column, and washed with 25 mM sodium phosphate buffer pH 7.5, 5% glycerol, 0.5 mM EDTA, 2 mM DTT, and a linear NaCl gradient ranging from 0 to 1000 mM. Following the heparin column, the fractions containing the proteins were concentrated to 0.5 mL in a buffer containing a mobile phase with 25 mM sodium phosphate buffer pH 7.5, 300 mM NaCl, 1 mM EDTA, 3 mL DTT, 5% glycerol and then loaded onto a Superdex 200 Increase 10/300 GL. The final protein samples were snap‐frozen for storage.

### 2.4 Site‐Directed Mutagenesis

In this study, two C‐terminal deletion mutants of AtHelicase lacking the last 10 and 20 amino acids were constructed based on structural modeling of AtTwinkle, which indicates that its terminal 20 residues are highly flexible and potentially mediate interactions with the organellar replicative DNA polymerase. This predicted interaction is reminiscent of the well‐characterized interface between the T7 primase‐helicase and T7 DNA polymerase at the leading strand (Hamdan et al. 2005; Zhang, Lee, Zhu, et al. 2011; Gao et al. 2019). Mutagenesis was performed in the AtHelicase background to avoid confounding effects from the primase domain of AtTwinkle, which is known to interact with AtPolls and could complicate downstream analyses (Morley et al. 2019). Point and deletion mutants of recombinantly expressed AtTwinkle and AtHelicase proteins were generated in *E. coli* using the Q5 site‐directed mutagenesis protocol (E0554S, New England Biolabs). Briefly, a set of primers harboring the desired mutation was used to amplify the pCRI1b‐AtTwinkle or pET28‐SUMO‐AtHelicase plasmids. Typical 50 μL PCR reactions harbor 10 ng of plasmid DNA, 200 μM dNTPs, 0.5 μM of each primer, and 0.5 U Q5 High‐Fidelity DNA Polymerase. Reactions were incubated for 45 s at 98°C followed by 28 cycles of 98°C for 10 s, 57°C for 20 s, and 72°C for 160 s. PCR products were treated with kinase‐ligase‐DpnI mix (KLD mix) for 2 h at room temperature. 1 μL of the treated PCR product was used to transform chemically competent *E. coli* cells. Mutagenesis was corroborated by Sanger sequencing. Primers used in this study to carry out mutagenesis are listed in Table S1.

### 2.5 Substrate Assembly for DNA Unwinding

DNA helicase activity of recombinantly expressed proteins was measured using a forked DNA substrate that involved combining different oligonucleotides (Tables S2 and S3). Substrates were prepared at 500 nM, with a 10% excess of non‐labeled oligos in a reaction buffer containing 10 mM Tris–HCl pH 8.0, 1 mM EDTA, 150 mM NaCl. The reaction was heated to 95°C for 5 min and then allowed to cool slowly to room temperature. Proper substrate assembly was confirmed by native 10% polyacrylamide gel electrophoresis using 0.5× TBE (44.5 mM Tris base, 44.5 mM Borate, 1 mM EDTA pH 8.0) as the running buffer. Assembled substrates were then recovered by electroelution from excised gel slices in 1× TBE (89 mM Tris base, 89 mM Borate, 2 mM EDTA pH 8.0) containing 10 mM MgCl_2_, followed by dialysis against TE buffer (10 mM Tris–HCl pH 8.0, 1 mM EDTA). Primer‐template extension and strand‐displacement substrates were assembled using the oligonucleotides listed in Tables S2 and S3.

### 2.6 Primer Extension and Strand‐Displacement Reactions by AtPolls

Primer‐extension and strand‐displacement reactions were carried out in a buffer containing 20 mM Bis‐Tris‐propane (pH 7.2), 100 mM monopotassium glutamate, 10 mM MgCl_2_, 10 mM DTT, and 0.2 mg L^−1^ BSA. The primer‐extension reactions were accomplished by adding 3 nM of pGEM‐3Zf(+), 5 mM of AtPolIA, and 0.1 mM of dNTP's. For the strand‐displacement reactions, we added 25 nM synthetic replication fork (see Table S3), 5 nM of AtPolIA and 480 nM of AtHelicase/AtTwinkle, 1 mM ATP and 0.2 mM dNTP's. All the reactions were incubated at 37°C for the durations specified in each figure. After incubation, samples were run in a 0.8% agarose gel and 15% acrylamide gel, respectively, for primer‐extension and strand‐displacement reactions. Gels were analyzed by scanning for fluorescence on a GE Typhoon Gel scanner.

### 2.7 DNA Polymerase Activity Stimulation Assay

The typical reaction mix contained 7 nM of a modified single‐stranded PGEM‐3Zf (+) DNA (3197 nucleotides), provided by Professor Juan C. Alonso, with an annealed 60‐nucleotide primer (Tables S2 and S3). The reaction also included 55 nM of wild‐type AtPolIA, 660 nM of the Helicase domain, and 100 μM dATP. The reaction buffer consisted of 20 mM Bis‐Tris‐propane (pH 7.2), 100 mM potassium glutamate, 1 mM DTT, and 0.2 mg mL^−1^ BSA. AtPolIA and AtHelicase were mixed and incubated on ice for 5 min and then added to a mix containing the reaction buffer and dATP or ATP to allow DNA polymerase assembly. This mixture was incubated for 15 min at room temperature. Reactions were initiated by adding a mix of 10 mM MgCl_2_ and 100 μM of each dNTP (dATP, dTTP, dCTP, dGTP). The reactions were incubated at 30°C and terminated at various time points by adding 50 mM EDTA (pH 8.0). Alkaline loading buffer (1×, 50 mM NaOH, 1 mM EDTA, and 3% (w/v) Ficoll) was then added to the reactions. The samples were loaded onto a 0.8% alkaline agarose gel, which was run at 20 V overnight (16 h) and scanned for fluorescence on a GE Typhoon Gel scanner in the FAM or Cy5 mode.

### 2.8 Helicase Assessment by an Electrophoretic Mobility Shift Assay

DNA helicase reactions were performed in a buffer containing 20 mM Bis‐Tris‐Propane (pH 7.5), 10 mM MgCl_2_, 10 mM DTT, 50 mM potassium glutamate, and 0.1 mg mL^−1^ BSA. The reactions used a fluorescently labeled substrate at a concentration of 5 nM, and AtTwinkle or AtHelicase at 100 nM. The 10 μL reactions were incubated at 37°C for 30 min and then stopped by adding an equal volume of 2× stop buffer (8% glycerol, 0.6% SDS, 40 mM EDTA). The samples were then loaded onto a 12% native acrylamide gel and run at 150 V for approximately 2 h at 4°C in 0.5× TBE (45 mM Tris Borate, 10 mM M EDTA, pH 8.3) until the bromophenol blue dye reached the bottom of the gel. The gels were scanned for fluorescence on a GE Typhoon Gel Scanner in the FAM or Cy5 mode.

The replication‐fork structures for the 5′ and 3′ strands were formed by annealing different oligos (Tables S2 and S3). These oligonucleotides would hybridize, forming a 28‐nucleotide double‐stranded region, leaving 3′ and 5′ overhangs.

Proper assembly of the substrates was corroborated via electrophoresis on a native 10% polyacrylamide gel using 0.5× TBE buffer. Following separation, assembled substrates were electroeluted from excised gel slices in 1× TBE supplemented with 10 mM MgCl_2_ and subsequently dialyzed against TE buffer.

### 2.9 Microscale Thermophoresis

For binding experiments, AtPollB was labeled using the NanoTemper's Monolith His‐Tag Labeling Kit. Fluorescent labeled DNA polymerase was maintained at a constant concentration, whereas AtHelicase and AtHelicase‐Δ10 were titrated using increasing concentrations. Binding assays were performed in PBS buffer supplemented with 0.05% Tween‐20 using a NanoTemper Monolith NT.115p instrument. Data were analyzed and fitted according to previously described methods (Peralta‐Castro et al. 2017).

### 2.10 Plant Materials and Growth Conditions

*Arabidopsis thaliana* ecotype Columbia (Col‐0) was used as the wild‐type plant throughout this study. The T‐DNA insertion mutant lines for the *AtTwinkle* gene were the *ph 1* line (SALK_152246) and the *ph 2* line (WiscDsLox423D2:CS855183), both obtained from corresponding T‐DNA insertion line collections. Seeds were surface sterilized by treatment with a solution of 5% sodium hypochlorite for 15 min and then were washed and imbibed in sterile water for 2 days at 4°C to obtain homogeneous germination. Seeds were sown on commercially available MS 0.5× agar medium and grown under short‐day conditions (8 h light, 16 h night, 21°C) in a growth chamber. For genotoxic assays, 4‐day‐old seedlings were transferred to fresh MS 0.5× agar medium without treatment or supplemented with the genotoxins ciprofloxacin (0.4 mM) or hydroxyurea (0.5 mM), where the root length was measured 10 days after transfer. Primary root length measurements were obtained using the ImageJ program.

### 2.11 Genotyping

The DNA was extracted using Edwards extraction buffer (200 mM Tris–HCl (pH 7.5) 250 mM NaCl, 25 mM EDTA, 0.5% SDS), and polymerase chain reactions (PCR) were performed to genotype *ph 1* and *ph 2* using the corresponding primer pairs indicated in Table S4. Because homozygous *ph 2 (−/−)* plants are embryo lethal, we decided to characterize *ph 2* (+/−) heterozygous plants to discard wild‐type plants from the performed analysis. At the end of each growth time, all the grown plants were genotyped.

### 2.12 Aborted Seed Number Determination

Mature, slightly yellow‐green, completely closed siliques were selected per plant, 5–6 were placed in a 4:1 ethanol: acetic acid solution overnight. The next day, the solution was replaced with 90% ethanol; as soon as the solution acquired a greenish color, it was changed to 70% ethanol. After 2 days, photos of the siliques were taken under a Leica M205FA stereoscopic microscope. Then, the total and aborted seed numbers were counted.

### 2.13 Determination of Organellar DNA Copy Number

Total genomic DNA was extracted from 12‐day‐old seedlings of Col‐0, *ph 1* and *ph 2* (+/−) as previously described in (Doyle and Doyle 1987). Organelle copy number was quantified by quantitative polymerase chain reaction (qPCR); the nuclear gene *UBC21* was used as a reference. Each qPCR reaction was performed using 1 ng of genomic DNA. For each organellar genome, we used two pairs of primers targeting two different genes (*COX1* and *CCMFN2* for the mitochondrial genome, and *RBCL* and *YCF2* for the chloroplastic genome). The primer sequences are listed in Table S4.

### 2.14 RNA Extraction and qRT‐PCR

Total RNA was extracted from whole 12‐day‐old plantlets using the NucleoSpin RNA protocol (Macherey‐Nagel, Germany Cat. # 740949). First‐strand cDNA was synthesized from 1 μg of total RNA by using the ImProm‐IITM Reverse Transcription System (Promega) according to the manufacturer's instructions. 1 μL of synthesized cDNA was mixed with 100 nM of each primer and 15 μL Maxima SYBR Green/ROX qPCR Master Mix (2×) from Thermoscientific. Products were amplified, and fluorescent signals were acquired with an Applied Biosystems 7500 detection system. The specificity of the amplification products was determined by melting curves. Experiments were performed in triplicates, and the presented data are representative of at least three biological replicates. The sequences of primers used in this study are provided in Table S4. Expression levels of the *TWINKLE* gene were normalized using the housekeeping gene *EMB2386*.

## 3 Results

### 3.1 AtTwinkle Is a Conserved Bifunctional Primase‐Helicase

**FIGURE 2:**
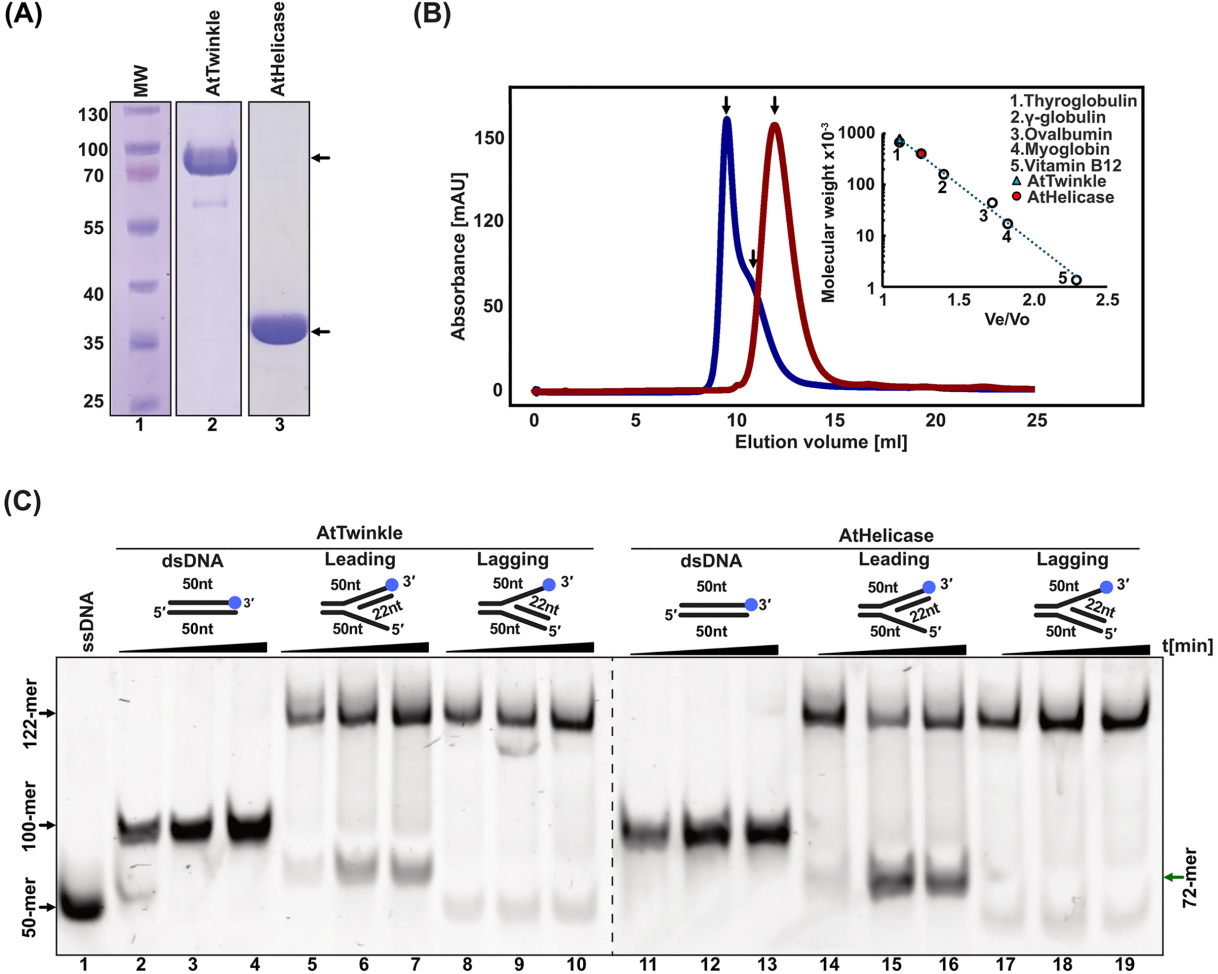
AtTwinkle is a functional hexameric replicative DNA helicase. (A) Heterologous purification of recombinant AtTwinkle and AtHelicase after three chromatographic steps. Purified protein samples were run onto a 10% SDS‐polyacrylamide gel stained with Coomassie Brilliant Blue. AtTwinkle and AtTwinkle have a theoretical mass of 70.12 and 35.3 kDa, respectively. (B) Oligomeric state of AtTwinkle and AtHelicase determined by size‐exclusion chromatography. Elution profiles of AtTwinkle (blue) and AtHelicase (red) run on a Superdex 200 gel filtration column calibrated with molecular weight standards: Thyroglobulin (670 kDa), γ‐globulin (158 kDa), ovalbumin (44 kDa), and myoglobin (17 kDa). Apparent molecular masses (Mr) are determined by plotting the elution volumes of the standards against the logarithm of their molecular weights. According to their M_r_ (relative molecular mass) both AtTwinkle and AtHelicase migrate as hexameric assemblies. (C) dsDNA unwinding by AtTwinkle and AtHelicase is limited to substrates with 5′–3′ directionality (Table S2). Time course (0, 15, and 30 min) DNA unwinding reaction using substrates with different or without overhangs. Product accumulation is only observed in substrates with 5′ overhangs.

To investigate the enzymatic activities of AtTwinkle, we utilized two constructs: one comprising the full‐length protein, harboring both primase and helicase activities, and another containing only the helicase domain (AtHelicase). Both proteins were purified to > 95% purity through immobilized metal affinity chromatography (IMAC), ion‐exchange chromatography, and gel filtration (Figure 2A). T7 primase‐helicase and human Twinkle assemble as donut‐shaped oligomers, existing in a range of oligomeric states from hexamers to nonamers. This oligomerization is essential for helicase activity, as it facilitates the assembly of a composite active site using adjacent subunits for nucleotide hydrolysis (Sawaya et al. 1999; Singleton et al. 2000; Toth et al. 2003; Ziebarth et al. 2010; Fernandez‐Millan et al. 2015; Riccio et al. 2022). Gel filtration analysis of purified AtTwinkle and AtHelicase in the absence of metal ions and nucleotides revealed that AtTwinkle elutes in two distinct peaks. The first peak corresponds to a very large complex (~800 kDa), while the second peak is similar to the elution profile of thyroglobulin and is consistent with the size of an AtTwinkle nonamer (~670 kDa). These results indicate that AtTwinkle, like its human counterpart, assembles into various oligomeric forms, likely adopting conformations that include closed and partially open ring‐shaped hexamers, nonamers, or decamers (Sawaya et al. 1999, Singleton et al. 2000, Toth et al. 2003, Jemt et al. 2011, Figure 2B). The oligomerization properties of AtTwinkle and AtHelicase are intrinsic and occur in the absence of substrates, resembling human Twinkle. This contrasts with T7 primase‐helicase, which requires the presence of metal ions, nucleotides, or DNA for oligomer formation (Dong and von Hippel 1996; Milenkovic et al. 2013). Bacteriophage T7‐like replicative hexameric helicases unwind DNA while translocating along the 5′–3′ strand and excluding the 3′ strand from its central channel. Hexameric helicases require a single‐stranded overhang to load onto forked DNA substrates (Lechner and Richardson 1983; Hingorani and Patel 1996). Helicase assays were performed in the presence of all NTPs using substrates that mimic replication‐fork structures with 3′, 5′, or no overhangs. These experiments demonstrated that AtTwinkle and AtHelicase are unable to translocate along DNA in the 3′–5′ direction (Figure 2C, lanes 5–7 and 14–16) or in the absence of a single‐stranded overhang (Figure 2C, lanes 2–4 and 11–13), but unwind synthetic forked DNA substrates in the 5′–3′ direction (Figure 2C, lanes 8–10 and 17–19). AtHelicase exhibits greater dsDNA unwinding activity compared to full‐length AtTwinkle, potentially due to enhanced stability of its hexameric assembly, as suggested by gel filtration analysis (Figure 2B). The independent helicase activity of AtHelicase suggests that this domain can be used to investigate protein–protein interactions between this module and AtPolls. The 5′–3′ translocation polarity observed for AtTwinkle and AtHelicase indicates that AtTwinkle likely interacts functionally with organellar DNA polymerases or other components of the plant organellar replisome, facilitating replication of the leading strand.

### 3.2 AtTwinkle dsDNA Unwinding Depends on ATP and dATP

**FIGURE 3:**
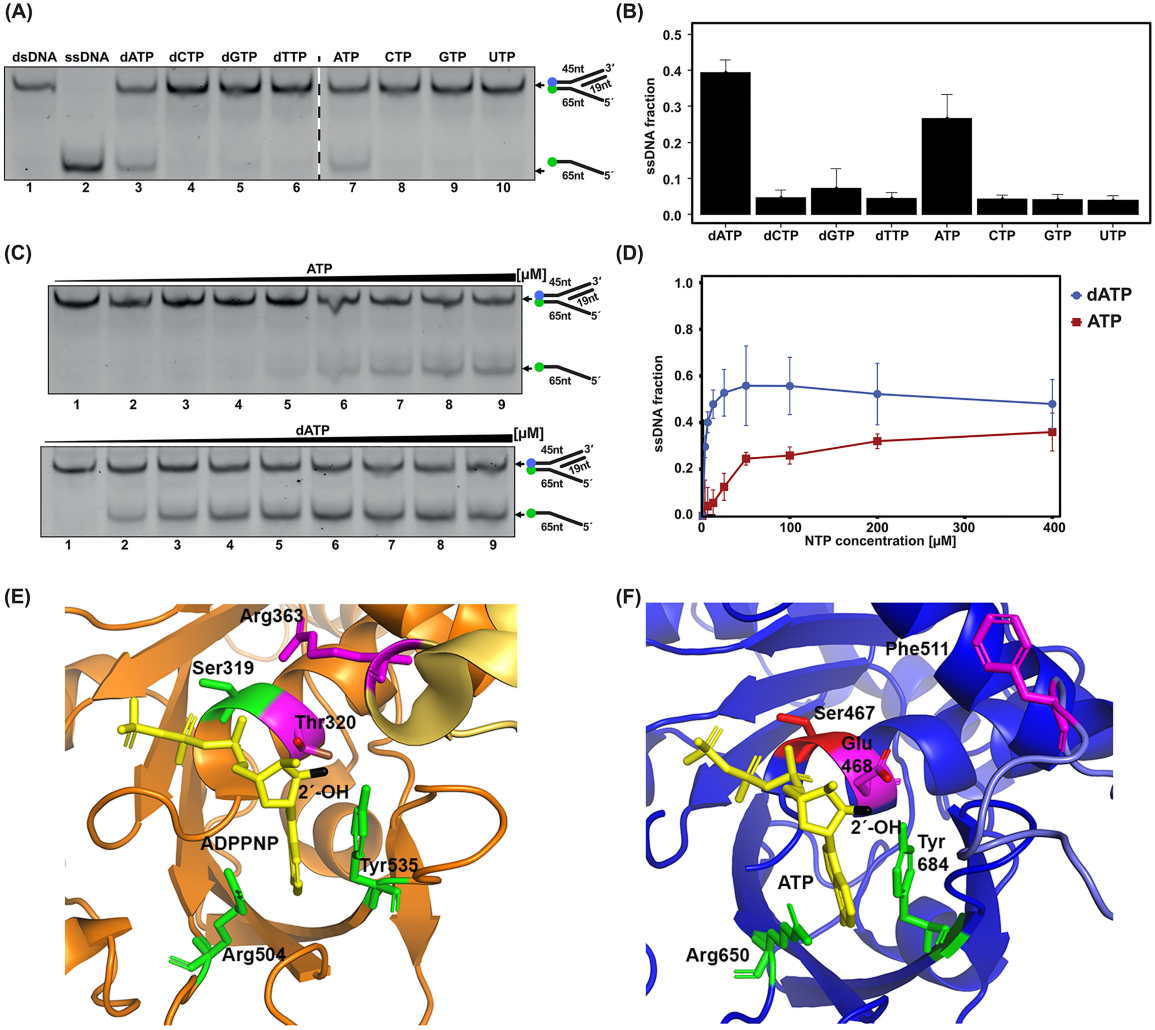
AtTwinkle is a fork‐specialized unwinding helicase with preference for ATP or dATP. (A) Unwinding of synthetic forked DNA substrate with 5′ overhangs (Table S2) by AtTwinkle in the presence of saturating concentrations (500 mM) of NTP or dNTPs. The dsDNA unwinding is shown on an 8% native polyacrylamide gel after an incubation period of 20 min. (B) Graphical representation of DNA unwinding by AtTwinkle using NTP or dNTPs. The relative amount of ssDNA is represented using data from three independent experiments. (C) Unwinding by AtTwinkle using ATP and dATP concentrations of 3.125, 6.25, 12.5, 25, 50, 100, 200, and 400 mM (lanes 2–9). The relative positions of the dsDNA and ssDNA labeled oligonucleotide are indicated in the 10% acrylamide gel. (D) Graphical representation of DNA unwinding by AtTwinkle at varying concentrations of ATP or dATP. The relative amount of single‐stranded DNA (ssDNA) is shown as the mean ± standard deviation from three independent experiments. (E) Crystal structure of the helicase domain of T7 primase‐helicase (PDB: 1E0J) illustrating that residues Arg504 and Tyr‐535 interact with ADPNP substrate (yellow). The 2′‐OH of ADPNP is colored black and the rest of the nucleoside in yellow. Residue Thr320 is colored in magenta and its OH group in red. (F) Structural model of the helicase active site of AtTwinkle in complex with ATP. The structural model shows the conservation of residue Tyr 684 and Arg 550 and the presence of residue Glu468 as a potential steric gate that blocks the use of ATP but potentiates the use of dATP as a cofactor.

Replicative DNA helicases utilize specific nucleotides for hydrolysis, yet it remains unclear whether AtTwinkle exhibits a preference for specific nucleoside 5′‐triphosphates to drive dsDNA unwinding (Matson and Richardson 1983; Korhonen et al. 2003). T7 primase‐helicase efficiently hydrolyzes both dTTP and dATP, with a preference for dTTP, while dCTP and dGTP are not utilized (Satapathy et al. 2011). Additionally, T7 primase‐helicase can couple DNA unwinding to DNA synthesis using rATP, although it shows minimal efficiency with other ribonucleotides (Hingorani and Patel 1996). In contrast, human Twinkle preferentially uses dATP, dGTP, UTP, and ATP to facilitate DNA unwinding (Sen et al. 2012). Unwinding experiments using forked DNA substrates demonstrate that AtTwinkle and AtHelicase, unlike other replicative helicases, exclusively utilize ATP or dATP for dsDNA unwinding, showing no activity in the presence of pyrimidine nucleotides, GTP, or dGTP (Figure 3A,B). Furthermore, AtTwinkle exhibits a preference for dATP over ATP to mediate DNA unwinding (Figure 3C,D). dATP supports DNA unwinding at concentrations as low as 3 mM, whereas ATP requires a 10‐fold higher concentration to achieve comparable unwinding activity (Figure 3D). Structural studies of T7 primase‐helicase complexed with 5′‐adenylyl β,γ‐imidodiphosphate (ADPNP) or dATP reveal that Arg504 forms hydrogen bonds with ATP, while Tyr535 engages in base stacking interactions (Singleton et al. 2000; Toth et al. 2003, Figure 3E). An AlphaFold structural model of AtTwinkle, compared to the T7 primase‐helicase crystal structure, indicates that conserved residues Arg650 and Tyr684 occupy positions equivalent to Arg504 and Tyr535 in T7 primase‐helicase. Both T7 primase‐helicase and HsTwinkle contain a small threonine residue (T7 primase‐helicase: Thr320 and HsTwinkle: Thr423) near the 2′‐OH of ADPNP, enabling ribonucleotide accommodation within the nucleotide‐binding site (Figure 3E and Figure S1). However, in AtTwinkle, the corresponding residue is a bulkier glutamic acid (Glu468; Figure 3F), which, akin to the steric gate mechanism in DNA polymerases, likely restricts ribonucleotide binding (Astatke et al. 1998). Given that AtTwinkle preferentially utilizes dATP over ATP to facilitate DNA unwinding, it is plausible that dATP levels, rather than ATP concentrations, play a regulatory role in DNA replication within plant organelles.

### 3.3 AtPolls Depend on AtTwinkle for Strand‐Displacement on Long‐Substrates

**FIGURE 4:**
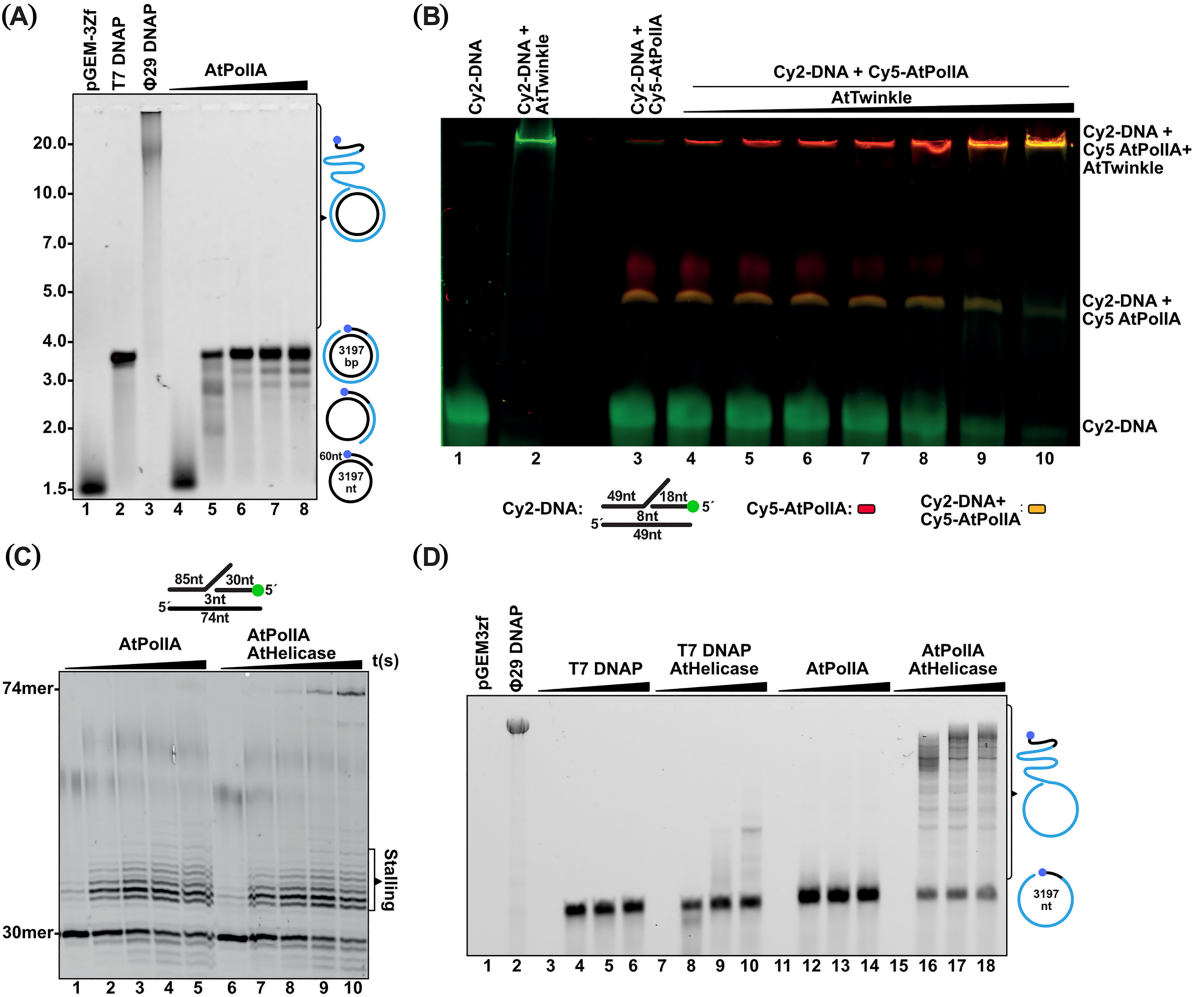
AtTwinkle functionally interacts with AtPolls for efficient DNA replication. (A) DNA synthesis on primed sscDNA substrate by AtPollA in comparison to T7 DNAP and phi29 DNAP. Reaction products were analyzed on 0.8% denaturing agarose gel. Extension reactions shown that AtPollA is inefficient for DNA unwinding at long circular DNA substrates. (B) Functional interaction of AtPoll‐AtTwinkle at a replication fork. Gel of a mobility shift assay showing the relative migration of a forked substrate in the presence of AtPollA and increased amount of AtTwinkle. (C) DNA polymerization‐coupled helicase assays on a synthetic replication fork. Primer‐extension reactions by AtPollA in the absence and presence of AtHelicase run on a 15% denaturing acrylamide gel. AtPolIA incorporates three nucleotides before encountering the double‐stranded DNA (dsDNA) region. Limited strand displacement allows for the incorporation of three to five nucleotides (lanes 2–5). Reactions performed in the presence of AtHelicase result in the accumulation of a 74‐mer full‐length product (lanes 7–10, upper band). (D) DNA polymerization‐coupled DNA unwinding on a long‐ssc plasmid substrate. Alkaline agarose gel showing rolling circle amplification by AtPollA in the presence of AtHelicase, in comparison to DNA amplification by AtPollA and T7 DNAP in the absence and presence of AtHelicase.

Most replicative DNA polymerases, with exceptions such as bacteriophage Phi29, yeast mitochondrial, and T5 DNA polymerases (Blanco and Salas 1996, Andraos et al. 2004, Viikov et al. 2011), require interaction with a DNA helicase to efficiently unwind long stretches of dsDNA. To evaluate the ability of AtPolls to unwind long dsDNA substrates, we assembled a primer‐template substrate by annealing a complementary oligonucleotide harboring a 5′‐tail of 35 unpaired thymines to a single‐stranded circular (ssc) DNA molecule of 3197 bp (pGEM‐3Zf (+); Promega). The resulting primer‐annealed ssc DNA migrated with a molecular mass corresponding to 1.5 kb of double‐stranded DNA (Figure 4A; Wanrooij et al. 2017, Wanrooij and Chabes 2019).

Primer‐extension reactions using this ssc pGEM‐primer substrate with T7 DNA polymerase (T7DNAP) produced an elongation product of approximately 3.5 kb, consistent with one round of polymerization on the substrate. This is characteristic of T7 DNAP, which lacks strand‐displacement activity and processively elongates until reaching the primer's 5′ end (Figure 4A, lane 2). In contrast, reactions with Phi29 DNAP resulted in elongation products exceeding 20 kb due to its robust strand‐displacement activity, enabling multiple rounds of elongation (Figure 4A, lane 3). Similar experiments with AtPollA yielded elongation products corresponding to one full round of elongation (Figure 4A, lanes 5–8). These results indicate that, like T7 DNAP, AtPolls lack strand‐displacement activity on long dsDNA substrates and therefore require interaction with a DNA helicase to achieve efficient dsDNA unwinding.

Although AtTwinkle physically interacts with AtPolls in the absence of a DNA substrate, the functional significance of this interaction remains unknown (Morley et al. 2019). To assess a potential functional interaction between AtTwinkle and AtPolls, we constructed a DNA substrate that mimics the topology of a replication fork by annealing three single‐stranded oligonucleotides (Figure 4B). The substrate comprises a 49‐nucleotide (49‐mer) template strand annealed to two complementary oligonucleotides: a partially complementary 49‐mer at the 3′ end and a Cy2‐labeled complementary 18‐mer at the 5′ end. This configuration generates a single‐stranded DNA (ssDNA) 5′‐tail which serves as a binding and translocation site for AtTwinkle and supports its 5′–3′ helicase activity. Additionally, the substrate permits 3′–5′ ′ movement of the replicative DNA polymerase (DNAP) along the template strand, enabling 5′–3′ nucleotide incorporation (Figure 4B, inset). When incubated with the DNA substrate, AtTwinkle and Cy5‐labeled AtPollA formed protein‐DNA complexes, which migrated to the top and middle portions of an 8% polyacrylamide gel, respectively (Figure 4B, lanes 2 and 3). Binding experiments with a constant concentration of Cy5‐labeled AtPollA and increasing amounts of AtTwinkle revealed two types of protein‐DNA complexes. At lower concentrations of AtTwinkle, a predominantly red‐colored band appeared, likely resulting from aggregation due to protein–protein interactions between AtTwinkle and AtPollA, along with a red‐colored band in the middle of the gel corresponding to AtPollA‐DNA complexes (Figure 4B, lanes 4–7). At higher concentrations of AtTwinkle, an orange band appeared at the top of the gel, corresponding to the forked substrate interacting with both AtTwinkle and AtPollA. This was accompanied by the disappearance of the red‐colored AtPollA‐DNA band in the middle of the gel (Figure 4B, lanes 8–10). These observations suggest the formation of a protein–protein complex involving AtTwinkle and AtPollA, potentially stabilizing their interaction with the forked DNA substrate.

### 3.4 Strand‐Displacement DNA Synthesis Mediated by AtTwinkle and AtPolls

Previous studies have shown that AtTwinkle interacts with AtPolls via its primase and helicase modules (Morley et al. 2019). To functionally investigate the interaction of AtTwinkle's helicase module during leading‐strand synthesis, we used its independently expressed helicase module (AtHelicase), as has been routinely assessed for the T7 DNA replication system (Lee and Richardson 2011; Satapathy et al. 2011; Zhang, Lee, Zhu, et al. 2011; Kulczyk et al. 2012). To determine whether AtHelicase unwinds dsDNA for trailing AtPolls, we assembled a replication‐fork substrate in which the labeled primer and the blocking oligonucleotide (harboring an unpaired 5′‐tail) perfectly hybridize to the template strand, creating a gap of five nucleotides between them (Figure 4C). Reactions performed with AtPollA in the absence of AtHelicase resulted in the appearance of products that filled the gap between the primer and the blocking oligonucleotide, with only moderate strand displacement observed (i.e., elongation of 2–3 nucleotides beyond the blocking DNA strand; Figure 4C, lanes 1–5). The lack of nucleotide incorporation beyond the blocking oligonucleotide is consistent with the previously reported poor strand‐displacement activity of AtPollA (Trasvina‐Arenas et al. 2018). In contrast, reactions performed with an equimolar concentration of AtHelicase (as a hexamer) resulted in the appearance of full‐length products of 74 nucleotides, corresponding to complete primer extension (Figure 4C, lanes 6–15). To discern whether the full‐length product formation was due to a specific interaction coupling AtPollA polymerization with AtHelicase unwinding, we conducted a similar experiment using a single‐stranded circular (ssc) pGEM‐primer substrate. Reaction products were compared between T7 DNAP and AtPollA, both with equimolar amounts of AtHelicase. As observed, both T7 DNAP and AtPollA were unable to replicate beyond one round of nucleotide incorporation across the ssc plasmid in the absence of AtHelicase (Figure 4D, lanes 4–6 and 12–14). However, reactions incubated with T7 DNAP and AtHelicase resulted in the appearance of products corresponding to one round of replication, as well as products indicative of strand displacement after 1 and 2 h of incubation. By contrast, reactions with AtPollA and AtHelicase resulted in extension products corresponding to several rounds of DNA replication around the circular DNA substrate (Figure 4D, lanes 16–18). These results indicate that AtTwinkle selectively interacts with AtPollA, but not with other DNAPs, to couple DNA unwinding with DNA synthesis.

### 3.5 AtTwinkle Functionally Interacts With AtPols via a Positively Charged C‐Terminal Extension

**FIGURE 5:**
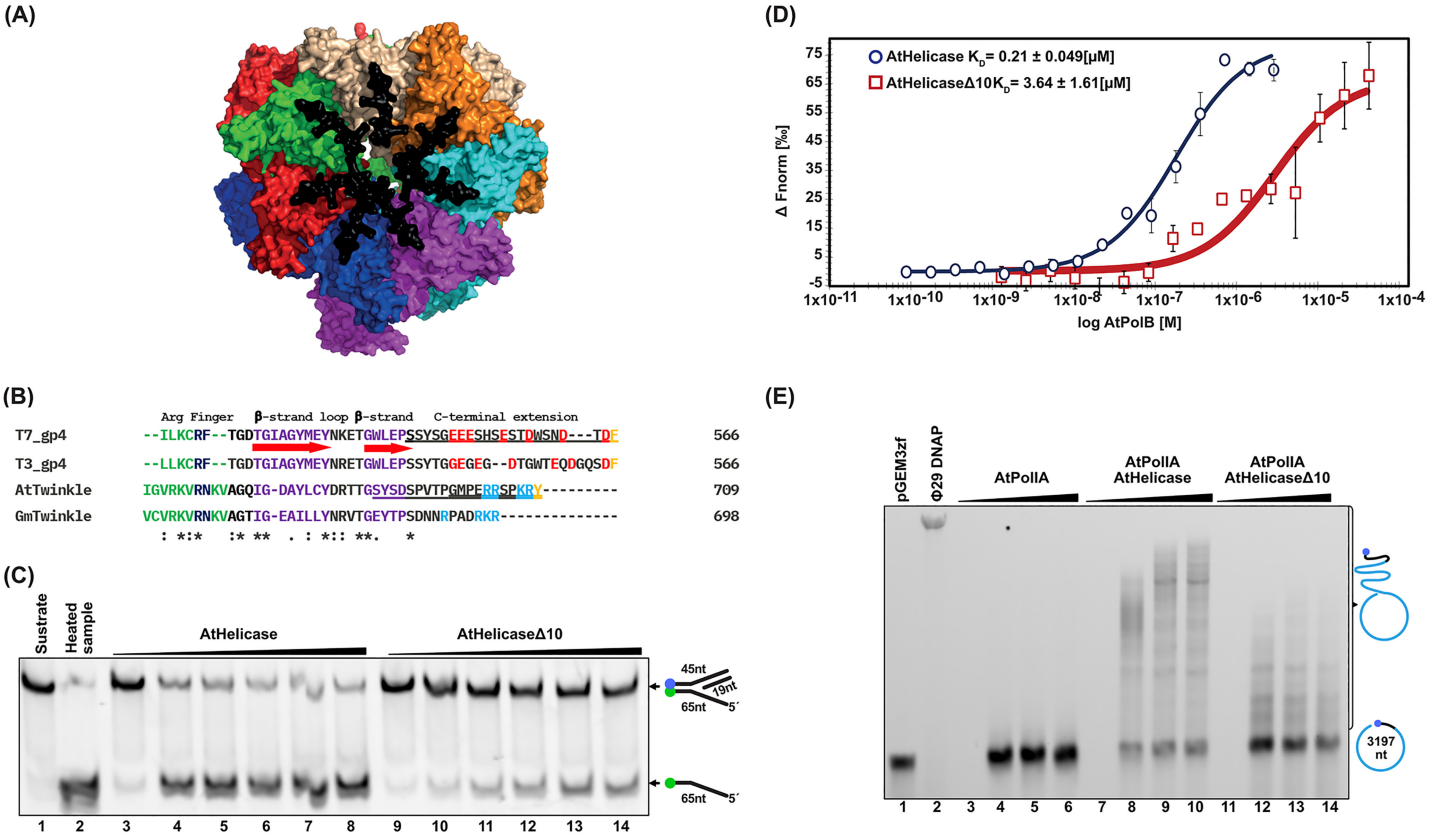
AtTwinkle and AtPolls interact via the C‐terminal extension of AtTwinkle. (A) Surface representation of structural model of AtTwinkle as a heptameric protein showing the localization of its positively charged C‐terminal extension. Each monomer of the modeled primase‐helicase is colored in a different color and the last 20 amino acids are colored in black. (B) Amino acid sequence alignment showing that AtTwinkle and T7 primase‐helicase share C‐terminal extensions with dissimilar characteristics. (C) DNA unwinding activities of AtHelicaseD10 in comparison to wild‐type AtHelicase. (D) AtHelicase‐AtPoll interaction in the absence of DNA assessed by microscale thermophoresis. The difference in normalized fluorescence is plotted against the logarithm of AtPollB concentration and fitted to a Langmuir isotherm. The Kds for the AtPollA interacting to AtHelicase and AtHelicaseD10 are 0.21 to 3.64 mM, respectively. (E) Coupled DNA helicase‐polymerization assay run on a 0.8% denaturing agarose gel using an ssc pGEM‐primer substrate.

AtTwinkle engages with AtPols via multiple functional domains, including the N‐terminal zinc finger domain, the central primase domain, and the C‐terminal helicase domain. The primase domain is thought to mediate interactions with lagging‐strand DNA polymerases, while the helicase domain primarily associates with the leading‐strand polymerase (Morley et al. 2019). AtTwinkle assembles into oligomeric helicase complexes and possesses a flexible C‐terminal region, comprising the final 20 amino acids, as suggested by structural modeling (Figure 5A). The C‐terminus of the T7 primase‐helicase, particularly the identity of its terminal phenylalanine residue and the acidic nature of its C‐terminal tail, is essential for interacting with two positively charged regions of the T7 DNAP (Hamdan et al. 2005; Zhang, Lee, Zhu, et al. 2011; Gao et al. 2019). These interactions, mediated by electrostatic interactions and aromatic forces, are critical for coordinating DNA unwinding with DNA synthesis. Structural alignments between bacteriophage and plant organellar Twinkles reveal the presence of an approximately 20‐amino‐acid C‐terminal extension in plant organellar Twinkles. Unlike the negatively charged C‐terminal extension in T7 primase‐helicase, the corresponding region in AtTwinkle is positively charged (Figure 5B). To investigate whether AtTwinkle and AtPolIs interact via the positively charged C‐terminal region of AtTwinkle, we utilized two deletion mutants of AtHelicase that remove 20 and 10 amino acids from the C‐terminus. The 20‐amino‐acid deletion mutant exhibited poor solubility and could not be purified (data not shown). In contrast, the 10‐amino‐acid deletion mutant (AtHelicaseΔ10) was readily purified and retained DNA helicase activity, albeit with reduced unwinding efficiency compared to the wild‐type enzyme (Figure 5C). Thermophoresis assays demonstrate that AtHelicaseΔ10 exhibits a ~25‐fold decrease in binding affinity for AtPolIB (from 0.21 to 3.64 μM), indicating that although the C‐terminal extension contributes to the interaction with AtPolIs, it is not solely responsible for AtPolI–AtHelicase binding. AtPolIB was used in these assays, as AtPolIA exhibits a pronounced tendency to dimerize, which complicates data interpretation (Garcia‐Medel et al. 2019; Figure 5D). Crucially, coupled DNA unwinding and DNA polymerization assays demonstrated that AtHelicaseΔ10 is less effective in coordinating DNA unwinding with AtPolIA‐mediated DNA synthesis (Figure 5E).

### 3.6 *AtTwinkle* Is an Essential Gene and the *Twinkle* M*utant Lines Display* a Decrease of the DNA Organellar Copy Number

**FIGURE 6:**
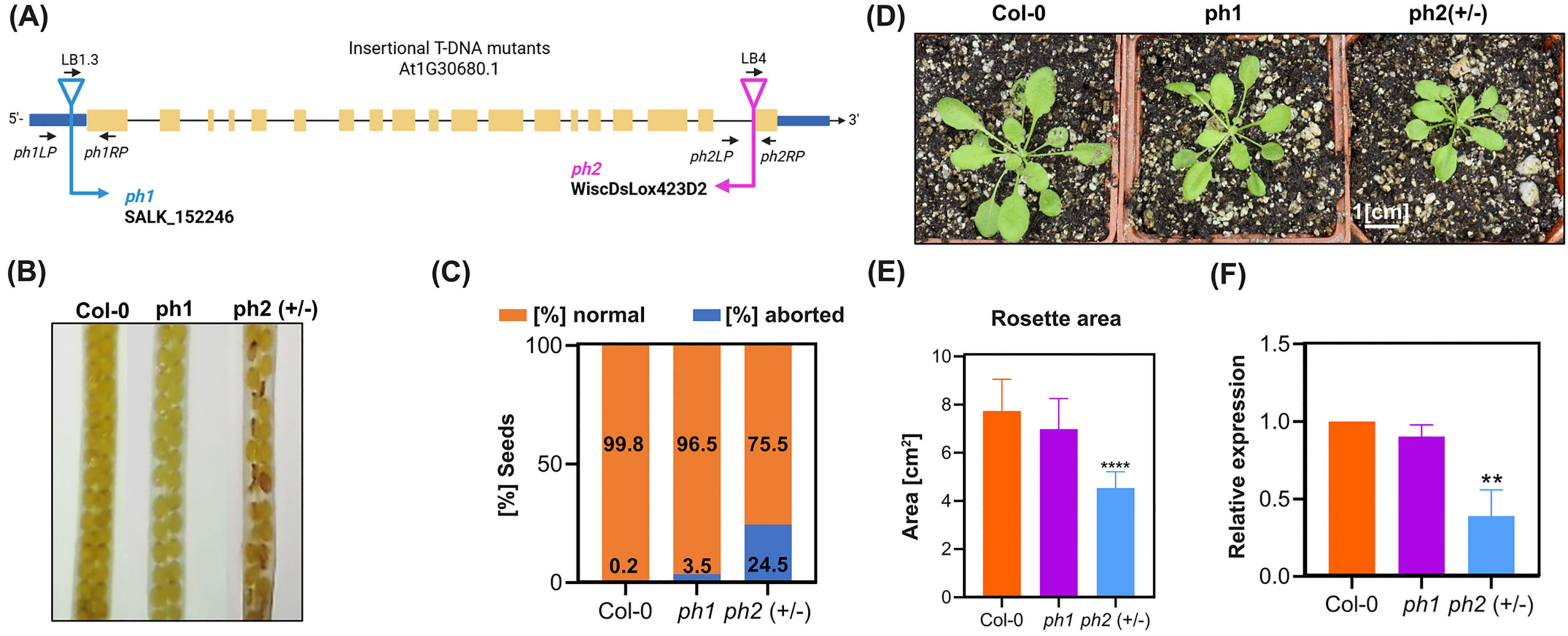
AtTwinkle is an essential gene in Arabidopsis. (A) Scheme indicating the T‐DNA insertion site in *ph 1* and *ph 2* lines of AtTwinkle. In the *ph 1* mutant, the T‐DNA insertion is located in the 5′ untranslated region (UTR), whereas in *ph 2*, the insertion occurs within the last intron. (B) Representative siliques of wild‐type, *ph 1* (−/−) and *ph 2* (+/−) plants, aborted seeds are observed in *ph 2* (+/−) siliques. (C) Percentage of normal and aborted seeds in wild‐type, *ph 1* and *ph 2* (+/−) siliques. (D) Phenotype for wild‐type, *ph 1* (−/−), and *ph 2* (+/−) plants. (E) Graphical representation of the rosette area of 4‐week‐old plants from Col‐0, *ph 1* and *ph 2* (+/−), bars are averages and standard deviations from 8 to 10 plants. (F) qPCR quantification of TWINKLE expression in mutants. The position of primer pairs is indicated in panel A. Data are presented as average +/− SD from three biological replicates with three technical replicates each one. The asterisks indicate significantly different values, between the mutants and Col‐0 as analyzed by Student's test (*) *p* < 0.0332, (**) *p* < 0.0021, (***) *p* < 0.0002, (****) *p* < 0.0001.

To further investigate the role of *AtTwinkle* (At1g30680) in organellar DNA replication in planta, two T‐DNA insertion mutant lines were analyzed. The *ph 1* line (SALK_152246) harbors a T‐DNA insertion in its 5′ UTR region (Figure 6A); this line was previously characterized and had a nearly wild‐type phenotype (Morley et al. 2019). The second mutant analyzed in this study, *ph 2* (WiscDsLox423D2:CS855183), carries a T‐DNA insertion within the 19th exon of the *AtTwinkle* gene, thereby disrupting the C‐terminal region of the protein (Figure 6A). Genotypic analysis of three self‐pollinated generations from *ph 2* plants revealed no homozygous mutants, suggesting lethality. Siliques from heterozygous *ph 2* (+/−) plants were examined and found to contain aborted seeds, unlike those from wild‐type and *ph 1* mutant plants, which displayed normal seed development (Figure 6B). Quantification of seed abortion showed that 24.5% of the seeds in *ph 2* (+/−) siliques were aborted (Figure 6C), consistent with Mendelian segregation for a recessive lethal allele. These findings indicate that homozygosity for the *ph 2* allele results in embryonic lethality, underscoring the essential role of *Twinkle* in seed viability. Notably, *ph 1* (−/−) mutants did not exhibit seed abortion, highlighting phenotypic differences between the two alleles. Consequently, subsequent analyses were focused on *ph 2* (+/−) and *ph 1* (−/−) plants.

The phenotype of four‐week‐old plants of *ph 1 (−/−)* was indistinguishable from wild‐type Col 0 (Figure 6D); in contrast, the heterozygous *ph 2* line displayed a retarded growth phenotype (Figure 6D), which was observed with a minor size of rosette (Figure 6E). Additionally, the *Twinkle* transcript levels were measured in *ph 1* (−/−) and *ph 2* (+/−) on 12‐day‐old seedlings using primers located after T‐DNA insertion in the *ph 2* mutation (Table S4). *Ph 1* (−/−) plants did not exhibit any significant differences compared to wild‐type plants; in contrast, *ph 2* (+/−) seedlings showed a reduced level of *Twinkle* transcripts (Figure 6F), suggesting that the *ph 2* allele affects the accumulation of the full‐length *Twinkle* mRNA. Consequently, the observed reduction in rosette area in *ph 2* (+/−) plants may be due to decreased *TWINKLE* expression or the production of a transcript encoding a defective AtTwinkle protein, which could impair mitochondrial DNA replication.

**FIGURE 7:**
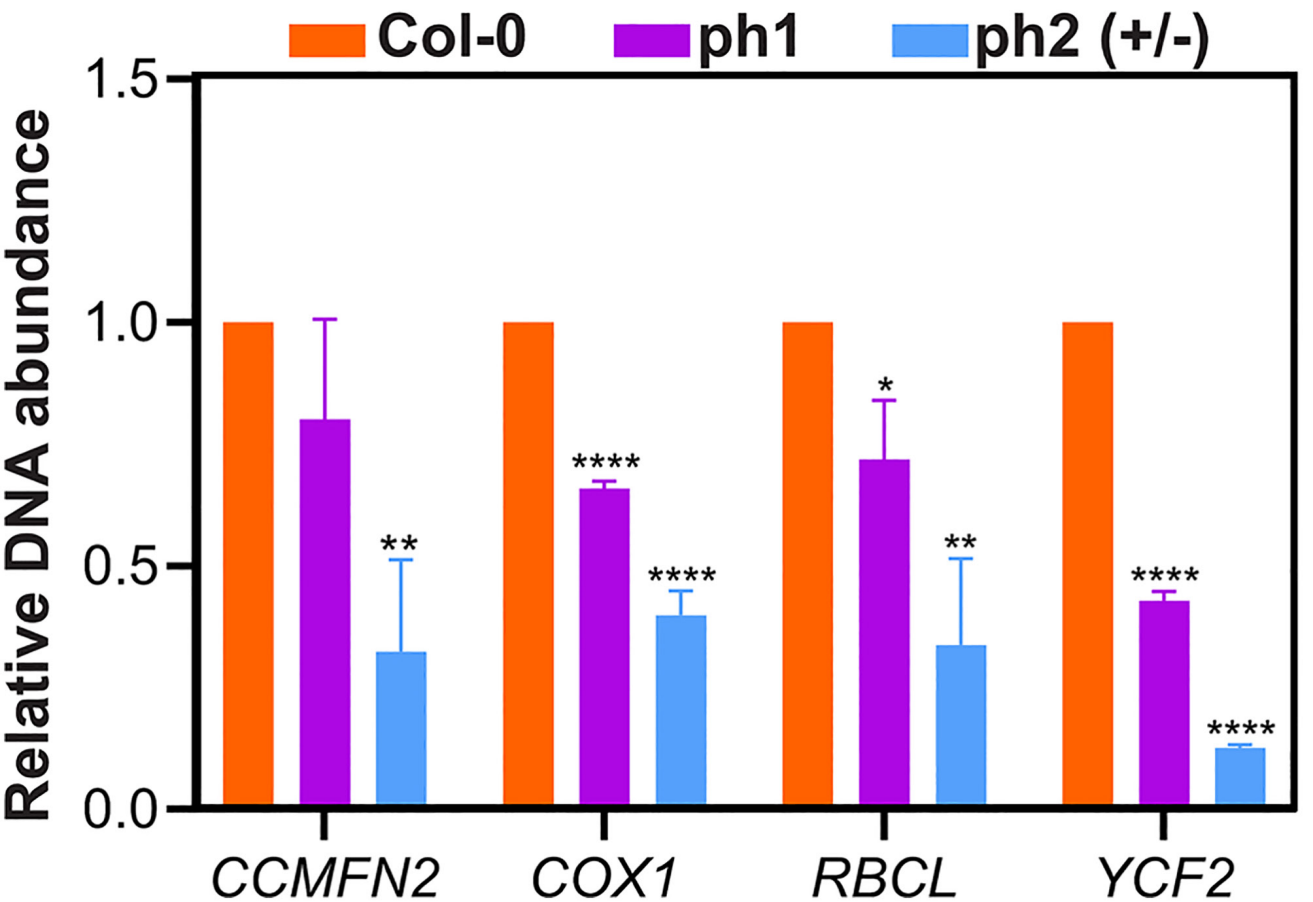
*Twinkle* mutants *ph 1* (−/−) and *ph 2* (+/−) exhibit a reduced abundance of organelle DNA abundance. qPCR analysis of mitochondria and chloroplast genome copy number relative to nuclear copy number in 12‐day‐old seedlings of Col‐0, *ph 1* (−/−) and *ph 2* (+/−). Total genomic DNA was extracted from 12‐day‐old seedlings of the indicated genotypes. Relative DNA copy number was measured by qPCR using the nuclear *UBQ10* gene as a reference; The *CCMFN2* gene, encoding a protein involved in cytochrome c biogenesis, and the *COX1* gene, encoding the main subunit of the respiratory complex IV, were used to monitor mitochondrial genome abundance, and the *YCF2* (encoding a protein of unknown function) and *RBCL* (encoding the large subunit of RuBisCO) genes were used for the chloroplast genome. Values are given as relative abundances compared with the wild type (Col‐0). Bars are averages and standard deviations, obtained from three technical replicates, and are representative of three biological replicates with three technical replicates each one. The asterisks indicate significantly different values, between the mutants and Col‐0 as analyzed by Student's test (*) *p* < 0.0332, (**) *p* < 0.0021, (***) *p* < 0.0002, (****) *p* < 0.0001.

AtTwinkle is predicted to be the only plant organellar DNA helicase; thus, it is expected that mutant lines display a reduced capacity to synthesize and accumulate organellar DNA. Therefore, it was compared to the organelle genome copy number in *twinkle* mutants and wild‐type 12‐day‐old plants. As shown in Figure 7, the relative abundance of organellar genomes compared with the nuclear genome was reduced in *ph 1*(−/−) as *ph 2* (+/−) in comparison with wild‐type plants. Overall, *ph 2* (+/−) seedlings showed a reduction, 50% higher in the relative abundance of the chloroplast genome compared to the mitochondrial genome (Figure 7). These results indicate that *ph 1* (−/−) contains fewer organelle genome copy numbers despite not showing phenotypical differences with respect to wild‐type plants. The decreased abundance of organellar genomes in the heterozygous *ph 2* (+/−) mutant suggests significant impairments in organellar DNA replication, which may be associated with the observed developmental defects.

### 3.7 *Twinkle* Mutants Are More Sensitive to DNA Damage in Mitochondria and Chloroplasts

**FIGURE 8:**
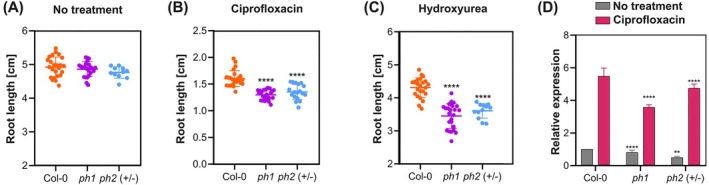
*Twinkle* mutants *ph 1 (−/−)* and *ph 2 (+/−)* show sensitivity to organellar DNA damage. Root length of Col‐0 and *twinkle* mutant lines germinated on MS 0.5X agar and transferred at day 4 to medium with or without genotoxins (0.4 μM CIP or 0.5 mM HU). (A‐C). Root length was measured 10 days after transfer. *n* = 24 for Col‐0 and *ph 1* (−/−) and for *ph 2* 8 (+/−), *n* = 11 in (A), *n* = 18 in (B) and *n* = 12 in (C). The asterisks indicate significantly different values, between the mutants and Col‐0 as analyzed by *t*‐test of unpaired data and comparison of means (*) *p* < 0.0332, (**) *p* < 0.0021, (***) *p* < 0.0002, (****) *p* < 0.0001. (D) Ciprofloxacin treatment induces an upregulation of the *TWINKLE* transcript. Col‐0 and *twinkle* mutant lines were grown on 0.5× MS agar medium for 9 days, then transferred to media either without or with 0.4 μM ciprofloxacin (CIP). After 2 days of treatment, whole seedlings were harvested for *TWINKLE* transcript analysis. Bars are averages and standard deviations obtained from three biological replicates with three technical replicates each one.

DNA‐synthesis activity is required for the repair of DNA lesions by mechanisms such as homologous recombination (HR). To evaluate the role of Twinkle in responding to DNA damage, we challenged seedlings of wild‐type and *twinkle* mutants to treatments with ciprofloxacin, which specifically inhibits the organellar gyrase, inducing double‐stranded breaks (DSBs) in plant organellar DNA (Evans‐Roberts et al. 2016). Furthermore, we also tested treatment with hydroxyurea (HU), which inhibits nucleotide synthesis leading to replication stress, fork collapse, and DSBs if the fork is not resumed properly. In contrast to ciprofloxacin, hydroxyurea induces replication stress in both the organellar and nuclear genomes. DSBs are repaired by homologous recombination in plant organelles, and we expect that *twinkle* lines that decrease the amount of available DNA molecules for HR would be especially susceptible to both genotoxins. To perform the assays, 4‐day‐old seedlings grown on MS 0.5X agar medium were transferred to medium supplemented with CIP (0.4 μM) or HU (0.5 mM); the root length was measured after 10 days of treatment. For the analysis of *ph 2* (+/−) plants, assays were performed on the progeny derived from heterozygous individuals. Following the experiment, all seedlings were genotyped, and only confirmed *ph 2* (+/−) plants were included in the final analysis. In the absence of genotoxins, the *ph 1* (−/−) and *ph 2* (+/−) mutants did not display differences in the root length in comparison to wild‐type plants (Figure 8A). Nevertheless, both *ph 1* (−/−) and *ph 2* (+/−) mutants displayed an increased sensitivity to ciprofloxacin and hydroxyurea (Figure 8B,C). Additionally, we sought to investigate whether the *AtTwinkle* gene is transcriptionally regulated in response to organellar DNA damage; 9‐day‐old plantlets were transferred to medium MS 0.5X supplemented with CIP 0.4 μM, after 2 days on treatment, *AtTwinkle* mRNA levels were determined in Col‐0, *ph 1*, and *ph 2* (+/−) plants. *AtTwinkle* mRNA was up‐regulated in the three lines; however, in *twinkle* mutants, the *AtTwinkle* transcript accumulation was minor in comparison to wild‐type plants (Figure 8D). Overall, our results suggest that the upregulation of AtTwinkle is essential for an appropriate response to organellar DNA damage.

## 4 Discussion

**FIGURE 9:**
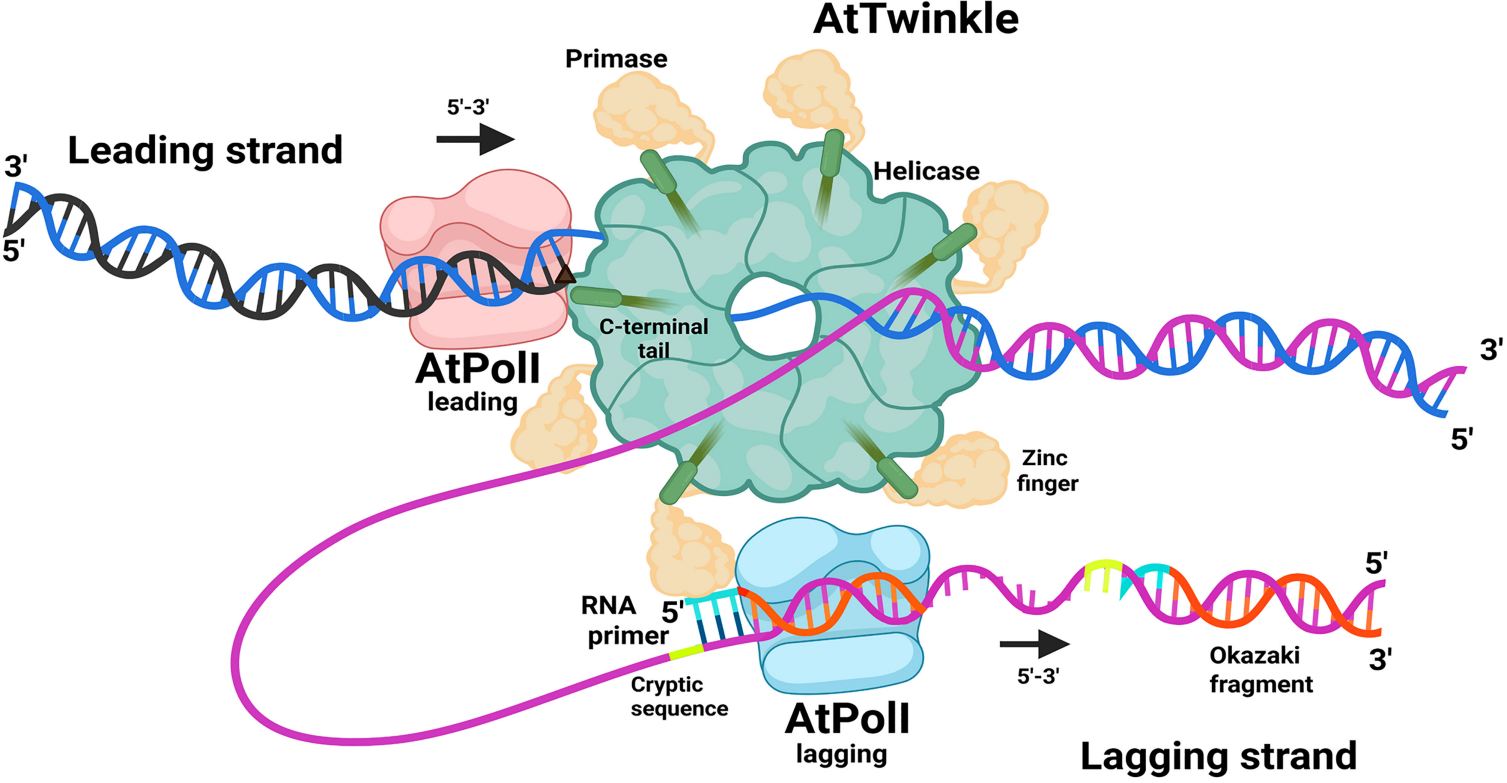
Structural model of the plant replisome of mitochondria and chloroplasts. AtTwinkle unwinds double‐stranded DNA (dsDNA; depicted in marine blue and purple), providing templates for both leading and lagging‐strand synthesis. The leading strand (marine blue) is used by the replicative AtPolI, which incorporate nucleotides in the 5′–3′ direction (indicated in black). The leading‐strand AtPolI interacts with the helicase domain of AtTwinkle via its C‐terminal positively charged extension, in a manner analogous to bacteriophage T7 replication. On the lagging strand, the primase domain of AtTwinkle synthesizes RNA primers (cyan), at a specific template sequence which includes two cryptic nucleotides (yellowing green; Peralta‐Castro et al. 2017). Primer hand‐off on the lagging strand is facilitated by the zinc finger subdomain (ZFD) of AtTwinkle's primase module and the lagging‐strand AtPol1, enabling the synthesis of Okazaki fragments (orange; Peralta‐Castro et al. 2023).

Seminal work by Gao and coworkers elucidated the assembly of the bacteriophage T7 replisome, showing for the first time the structural arrangement in which a DNA primase‐helicase synthesizes primers for the lagging‐strand DNA polymerase while coupling DNA unwinding for the leading‐strand DNA polymerase (Gao et al. 2019). Here we show that the plant organellar replisome comprises a bacteriophage‐related primase‐helicase (Twinkle) and bacterial‐related plant organellar DNA polymerases (POPs; Figure 9). In contrast, animal mitochondrial replisomes are composed of enzymes phylogenetically related to T‐odd bacteriophage enzymes (Cha and Alberts 1986, Cermakian et al. 1996, Shutt and Gray 2006b, a, Yao and O'Donnell 2008, Gao et al. 2019). Uniquely, plant Twinkle functions both as a primase and a replicative DNA helicase, underscoring the closer resemblance of plant DNA replication mechanisms to those of bacteriophages, as opposed to animals, where Twinkle lacks primase activity. We demonstrate that *Arabidopsis thaliana* Twinkle (AtTwinkle) acts as a bifunctional primase‐helicase, driving leading‐strand DNA synthesis. Previous findings established that AtTwinkle synthesizes RNA primers by recognizing a conserved 5′‐(G/C)GGA‐3′ sequence, in the antisense DNA strand, and utilizing two nucleotides (5′‐GA‐3′) as cryptic elements, which are nucleotides that are not copied into the primer itself but are nevertheless essential for primase recognition (Peralta‐Castro et al. 2017). These RNA primers are specifically extended by plant organellar DNA polymerases, but not by other polymerases (Peralta‐Castro et al. 2023). Furthermore, the zinc finger subdomain within the primase domain of AtTwinkle plays a critical role in primer delivery, enabling the extension of RNA oligonucleotides by plant organellar DNA polymerases during lagging‐strand synthesis (Peralta‐Castro et al. 2023; Figure 9). In this study, we primarily focus on the helicase activity of AtTwinkle and its functional interplay with the DNA polymerase during strand synthesis. AtTwinkle acts as an efficient 5′→3′ DNA helicase, with this polarity positioning it, to effectively couple DNA unwinding at the replication fork. Similar to other replicative helicases, AtTwinkle requires a single‐stranded 5′ DNA overhang for loading, and forked DNA substrates represent its preferred binding and activity site. This preference is consistent with the substrate specificity observed for other replicative helicases, such as human Twinkle and bacteriophage T7 primase‐helicase. AtTwinkle only uses ATP and dATP to drive unwinding, indicating that DNA replication is possibly linked to the concentration of both nucleotides. AtTwinkle effectively unwinds double‐stranded DNA (dsDNA) at a dATP concentration of 3 μM. Additionally, AtPols exhibit a Michaelis constant (kM) for deoxynucleotide triphosphate (dNTP) incorporation ranging from 15 to 35 nM. This efficiency contrasts with replicate DNA polymerases (DNAPs), which require approximately 50‐fold higher dNTP concentrations for optimal catalytic activity (Furge and Guengerich 1997; Satapathy et al. 2010; Satapathy et al. 2011). In eukaryotic organelles, where deoxynucleotide triphosphate (dNTP) pools are typically in the micromolar range and nucleotide triphosphates (NTPs) in the millimolar range, enzymes capable of functioning at nanomolar dNTP concentrations offer a robust mechanism to sustain and resume DNA synthesis under conditions of limited dNTP availability (Nick McElhinny et al. 2010). The helicase activity of AtTwinkle aligns with the observation that plant organellar DNA polymerases (AtPolls) exhibit limited efficiency in strand displacement on long DNA substrates. Unlike the T7 primase‐helicase, AtTwinkle couples DNA unwinding with processive DNA replication mediated by AtPols. This coordination is facilitated by an extended C‐terminal region of AtTwinkle, which acts as the interaction interface with AtPols. Consequently, plant Twinkle establishes a functional interaction with a DNA polymerase of distinct phylogenetic origin during leading‐strand DNA synthesis (Shutt and Gray 2006a, Figure 9).

Because we could not obtain homozygous lines and the proportion of aborted seeds in heterozygous siliques (~25%) aligns with the expected Mendelian segregation, we conclude that AtTwinkle is an essential gene. This observation is consistent with previous findings by Zhang et al., which demonstrated embryonic lethality in the absence of AtTwinkle (Zhang et al. 2024). We used our heterozygous plants *ph 2* (+/−) and homozygous *ph 1* (−/−) to study the role of AtTwinkle in DNA metabolism, both DNA repair and replication. The lethality observed in the *ph 2* mutant may be due to a T‐DNA insertion that results in a C‐terminal truncation of the protein, potentially disrupting the helicase domain located in this region. Our in vitro activity assays demonstrated that the deletion of the last 10 or 20 amino acids in this region severely compromises Twinkle's function. The T‐DNA insertion is likely to disrupt the final exon of the *Twinkle* gene, leading to loss of function. Interestingly, although *ph 1* (−/−) mutants do not exhibit noticeable phenotypic differences under standard growth conditions compared to wild‐type plants, they show a reduction in organellar DNA copy number. As anticipated, *ph 2* (+/−) plants exhibited an even more pronounced decrease in organellar DNA levels. Moreover, exposure of *ph 1* (−/−) and *ph 2* (+/−) plants to ciprofloxacin and hydroxyurea leads to a decrease in root length, indicating genomic instability and susceptibility to genotoxic agents. This susceptibility might be due to a decrease in *Twinkle* transcript and that the amount of AtTwinkle gene product is crucial for DNA metabolism. Proteomic studies revealed that AtTwinkle is at least 4‐fold more abundant than AtPolls (Fuchs et al. 2020). It is also possible that the T‐DNA insertion created truncated or chimeric forms of the AtTwinkle that act as roadblocks and obstruct DNA replication and transcription (Hernandez et al. 2020). Our studies agree with a recent study that demonstrated that AtTwinkle is an essential gene product (Zhang et al. 2024).

Bacteriophage‐related mitochondrial DNAP (Polg) is exclusively present in opisthokonts (Harada et al. 2024). According to the “succession of organellar DNA polymerase model” proposed by Moriyama and coworkers, plant organellar DNA polymerases were present before the divergence between plants and opisthokonts (Moriyama et al. 2011). If the ancestral mitochondrial endosymbiont possessed a phage‐related DNA polymerase (DNAP) and a phage‐related primase‐helicase, how did a DNA polymerase of distinct phylogenetic origin come to replace the original T‐odd‐related DNAP? We hypothesize that this evolutionary replacement conferred significant advantages. Specifically, plant organellar DNA polymerases from *Arabidopsis thaliana* (AtPolIA and AtPolIB) exhibit the ability to efficiently bypass DNA replication‐blocking lesions, such as abasic sites and thymine glycol (Baruch‐Torres and Brieba 2017; Baruch‐Torres et al. 2020). Furthermore, plants have integrated the specialized *MutS* mismatch repair gene, *MSH1*, which mitigates the error‐prone nature of AtPols, ensuring high‐fidelity mitochondrial replication while permitting lesion bypass (Baruch‐Torres and Brieba 2017; Ayala‐Garcia et al. 2018; Wu et al. 2020). In sum, we demonstrate that the bacteriophage‐related primase‐helicase AtTwinkle plays a crucial role in assembling the plant organellar replisome and interacting with plant organellar DNA polymerases to coordinate DNA unwinding and synthesis. The findings highlight the evolutionary adaptation of these two proteins, originating from distinct sources, to work together in the complex process of DNA replication in plant organelles. Additionally, the lethality observed in AtTwinkle mutants and their increased sensitivity to DNA‐damaging agents further emphasize the essential function of this protein in maintaining genomic stability.

## Author Contributions

L.G.B. and J.A.P.G. conceived and designed the research, were responsible for funding acquisition, and wrote the manuscript. C.M.M.‐V., M.A.D.H., A.P.C., L.D.C.M., C.D.R., D.S.A., and J.D.M.‐G. performed experiments and analyzed data. H.H.U., C.D.Q., and R.G.G. purified proteins, tested their activity and growth, and analyzed the T‐DNA insertion mutation lines. S.d.F. and A.C.R. supervised plant growth and in vivo studies. All authors contributed to manuscript writing and assembling figures and read and approved the manuscript.

## Conflicts of Interest

The authors declare no conflicts of interest.

## Supporting information


**Table S1.** Oligonucleotides used for site directed mutagenesis.
**Table S2.** Oligonucleotides used to substrate assembly for primer extension and DNA unwinding reactions.
**Table S3.** Substrate assemblies for primer extension and DNA unwinding reactions.
**Table S4.** Oligonucleotides used for the analysis of plants.

**Figure S1.** Structural alignment of AtTwinkle in comparison to human Twinkle and T7 Primase‐helicase focusing on the conserved helicase motifs.
**Figure S2.** AlphaFold structural model of AtTwinkle, AtPollA, and AtPollB.

## Data Availability

No datasets were generated or analyzed during the current study. The data supporting the findings of this study are available in the Supporting Information of this article and from the corresponding author upon request.
