# Supplementary material for: A Bacteriophage‐Derived Primase‐Helicase Orchestrates Plant Organellar DNA Replication
Source: Physiol Plant. 2025 Jul 7;177(4):e70379. doi: 10.1111/ppl.70379 (PMC12230644; doi:10.1111/ppl.70379)
Supplement: Supplementary file 1 — Table S1. Oligonucleotides used for site directed mutagenesis. Table S2. Oligonucleotides used to substrate assembly for primer extension and DNA unwinding reactions. Table S3. Substrate assemblies for primer extension and DNA unwinding reactions. Table S4. Oligonucleotides used for the analysis of plants. [file PPL-177-e70379-s002.docx]

**Table S1. Oligonucleotides used for site directed mutagenesis**

| Construct | Oligonucleotide | Sequence |
| --- | --- | --- |
| HelicaseΔ10 | Delta10F | TAAGGATCCGGCTGCTAACAAAGC |
| HelicaseΔ10 | Delta10R | ACCCGGGGTCACCGGTGAA |
| HelicaseΔ20 | Delta10F | TAAGGATCCGGCTGCTAACAAAGC |
| HelicaseΔ20 | Delta19R | GCTACCGGTGGTACGATCA |

**Table S2. Oligonucleotides used to substrate assembly for primer extension and DNA unwinding reactions**

| **Oligo Name** | **Sequence (5→3)** | **Label** |
| --- | --- | --- |
| OligoA | ATAAATATTTTTTATTAATAATAGATCACCTTTCTTTCTCTTCTCCCCTT | 3'-Cy5 |
| OligoB | AAGGGGAGAAGAGAAAGAAAGGTGATCTATTATTAATAAAAAATATTTAT |  |
| OligoC | TTCCCCTCCTCTCCTTCCTTCCTGATCTATTATTAATAAAAAATATTTAT |  |
| OligoD | GGAAGGAAGGAGAGGAGGGGAA |  |
| OligoE | AAGGGGAGAAGAGAAAGAAAGG |  |
| OligoF | TTTTTTTTTTTTTTTTTTTTAGTCGTAATCCGACCTCGAGGCATTGTCAATGTGCATAGAGTCAT | 3'-Cy2 |
| OligoG | ATGACTCTATGCACATTGACATGCTTCAGATTCGTATTGTACACT | 5'-Cy5 |
| OligoH | AGTGTACAATACGAATCTG |  |
| OligoI | TTTTTTTTTTTTTTTTTTTTTTTTTTTTTTTTTTCGCCAGGGTTTTCCCAGTCACGAC | 5'-Cy5 |
| OligoJ | AGCTATGACCATGATTAC | 5'-Cy2 |
| OligoK | AGCTACCATGCCTGCACGAATTAAGCAATTCGTAATCATGGTCATAGCT |  |
| OligoL | CAACTGCTCCGATGCCTTACCGTGCTTAATTCGTGCAGGCATGGTAGCT |  |
| OligoM | ATTGGAAGTAGGGATAGTCCCGAACCTCGC | 5'-Cy2 |
| OligoN | AGTGCTTACACCTGCCGCATGATCACGGTACGAGCTTGCTTTAGGCGAGGTTCGGGACTATCCCTACTTCCAA | 3'-InvdT |
| OligoO | TTTTTTTTTTTTTTTTTTTTTTTTTTTTTTTTTTTTTTTTTTTTTAGCAAGCTCGTACCGTGATCATGCGGCAGGTGTAAGCAC | 3'-InvdT |

**Table S3 Substrate assemblies for primer extension and DNA unwinding reactions**

| **Figure** | **Composition** | **Structure** |
| --- | --- | --- |
| **2C** | **OligoA + OligoB** | 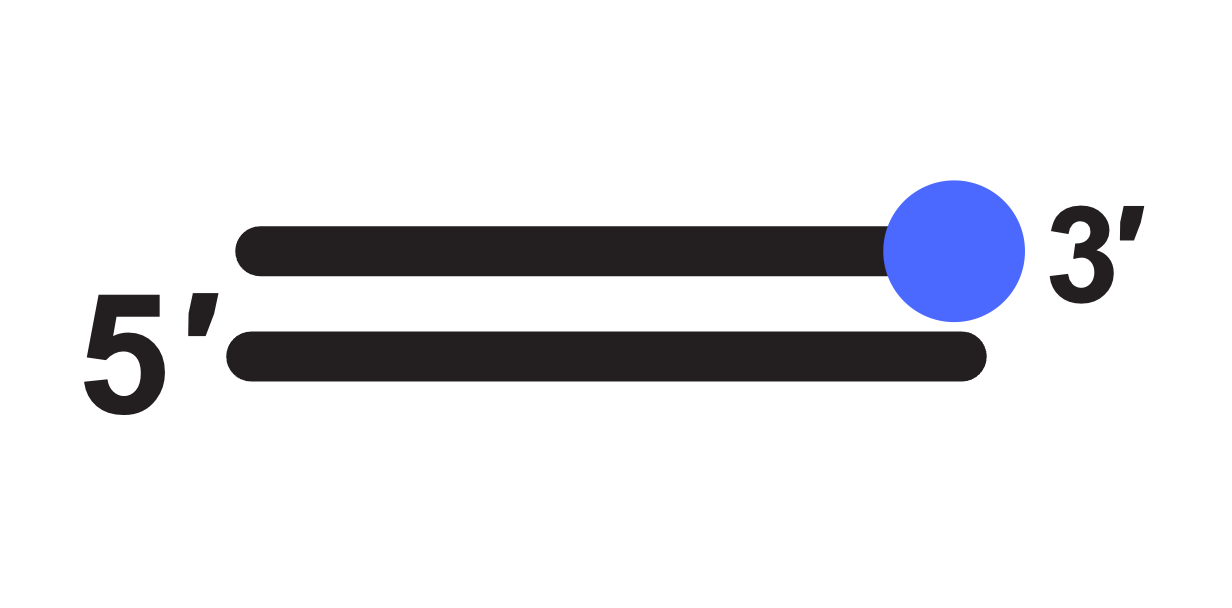 |
|  | **OligoA + OligoC + OligoE** | 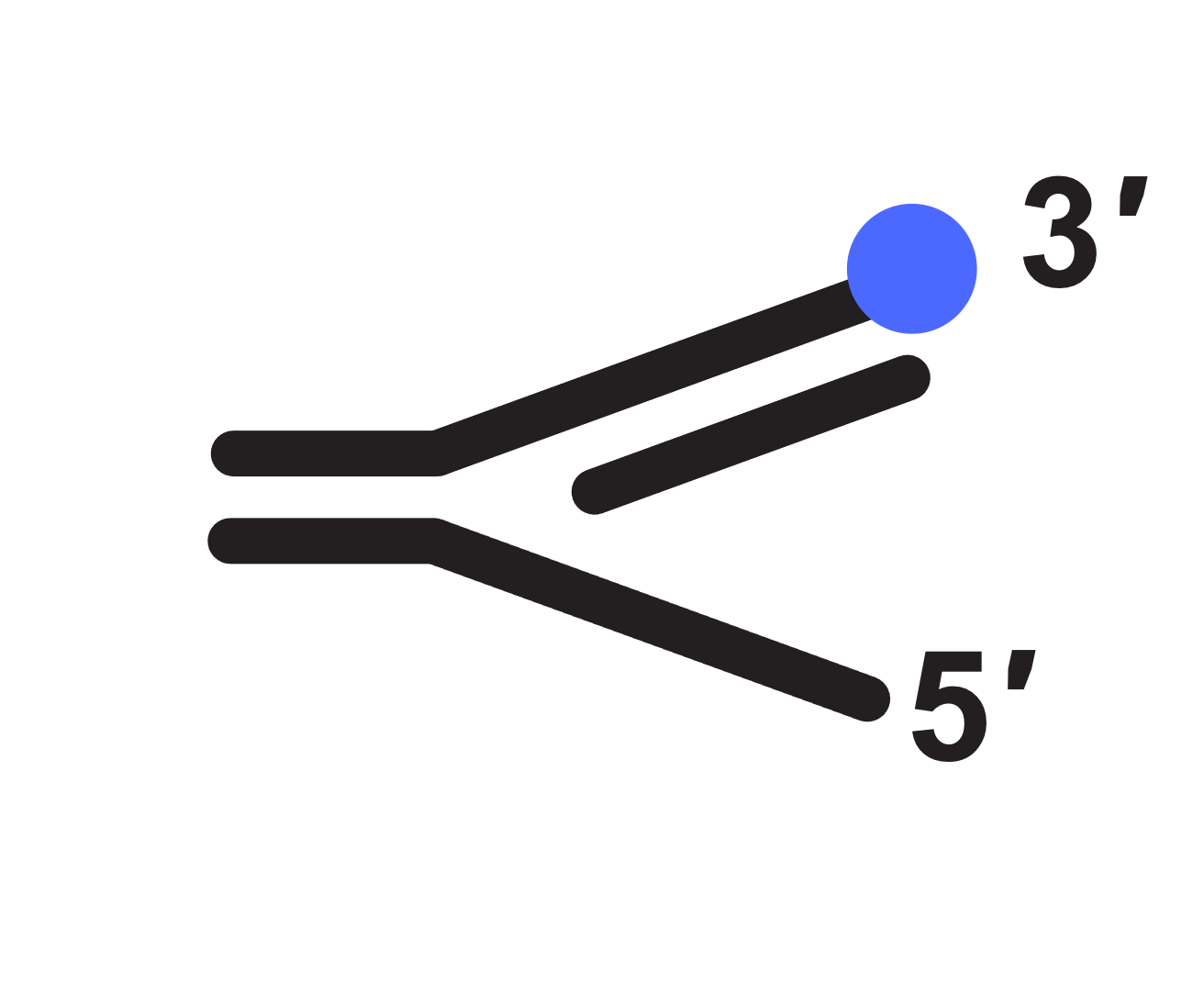 |
|  | **OligoA + OligoC + OligoD** | 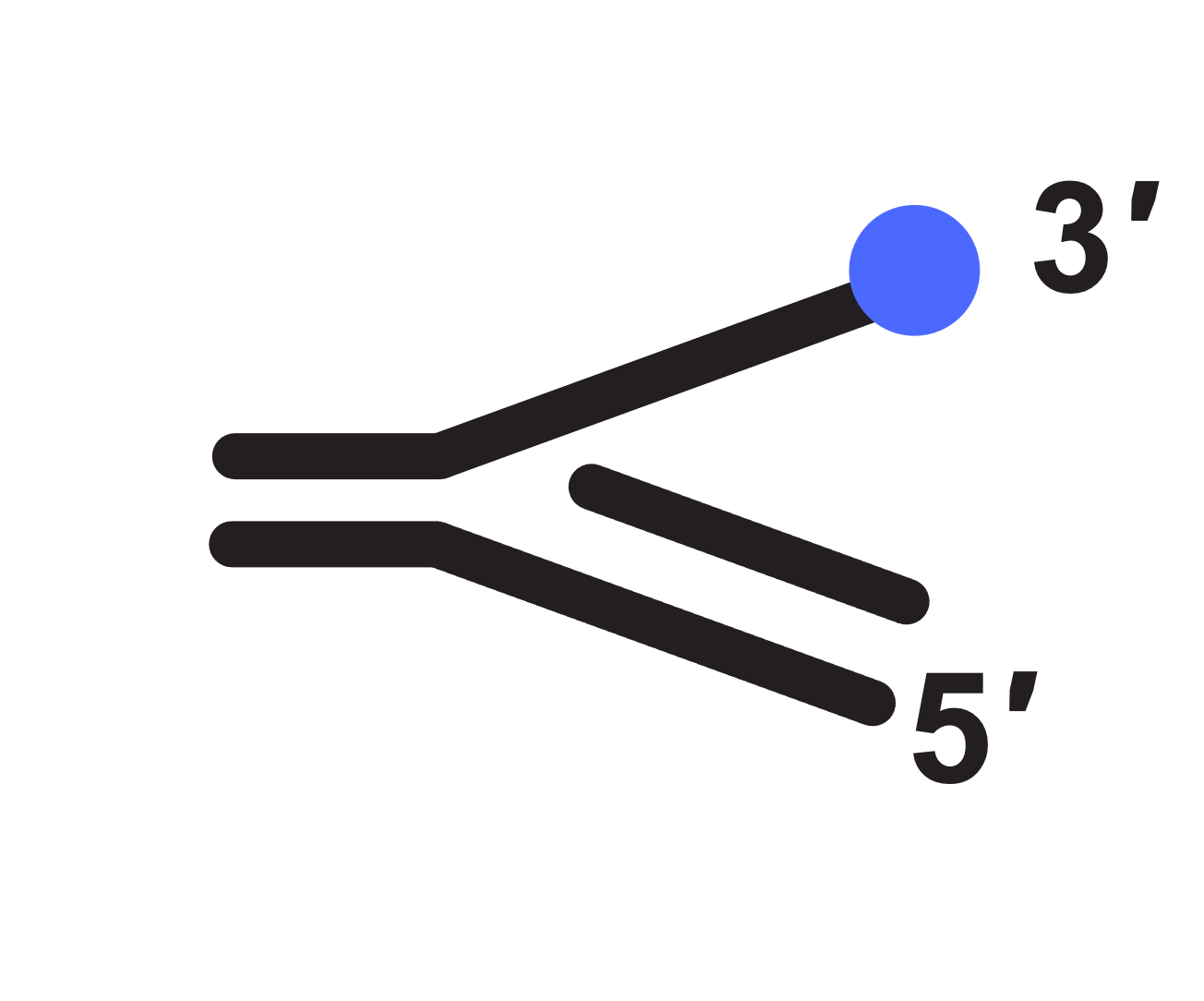 |
| **3A, 3C, 4C** | **OligoF + OligoG + OligoH** | 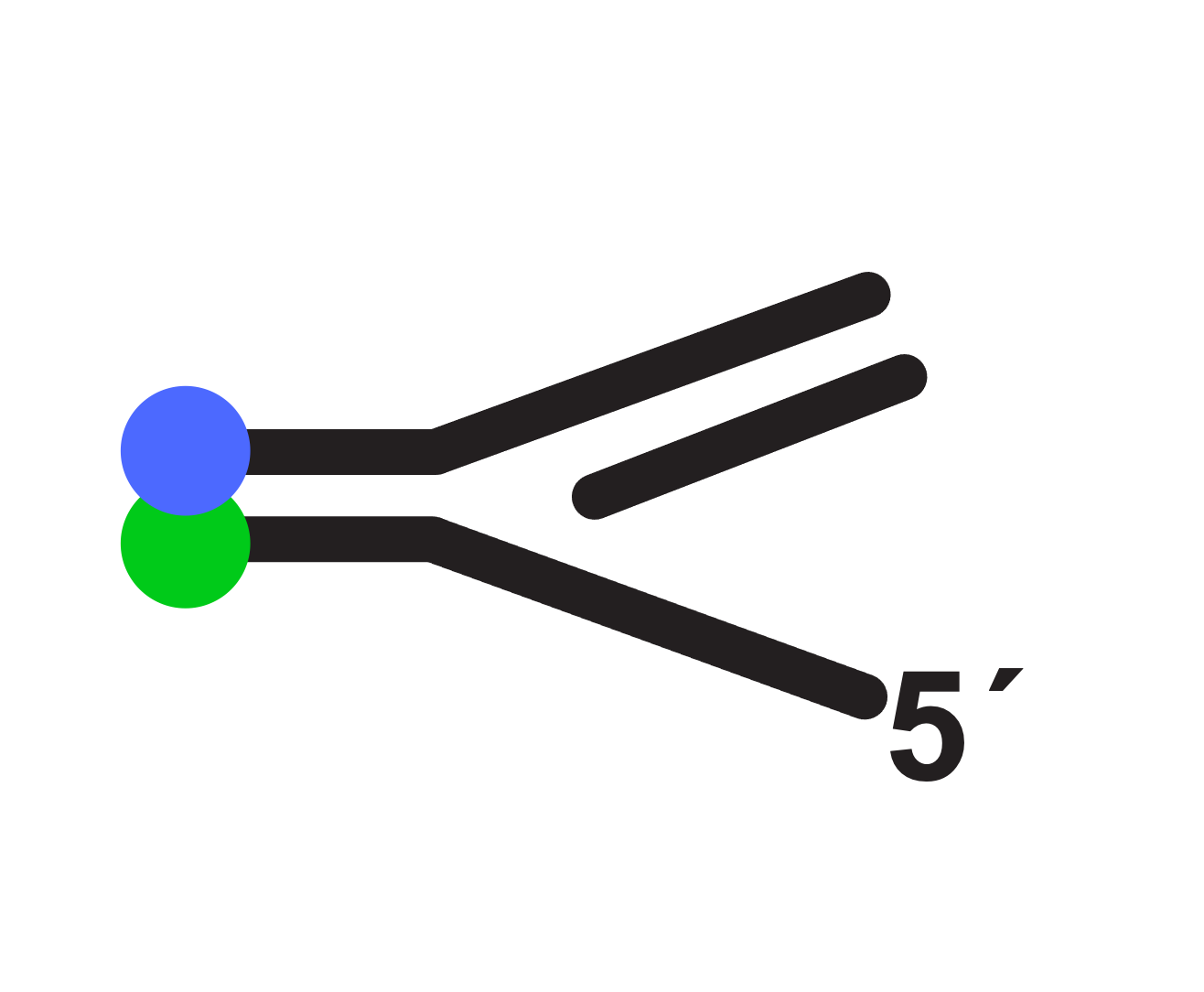 |
| **4E, 5A, 5D** | **pGEM-3Zf + OligoJ** | 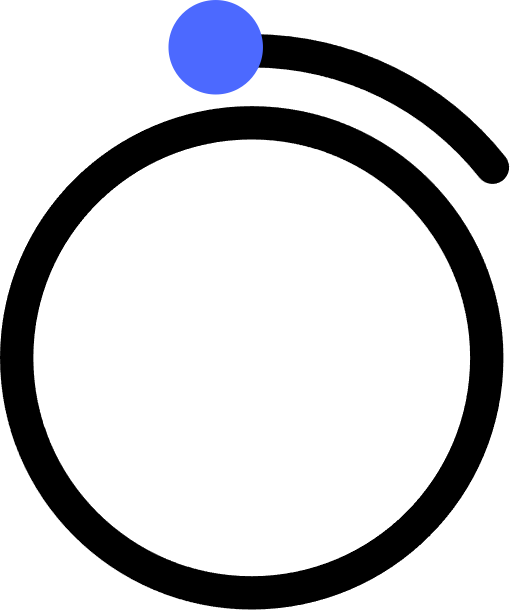 |
| **5B** | **OligoJ + OligoK + OligoL** | 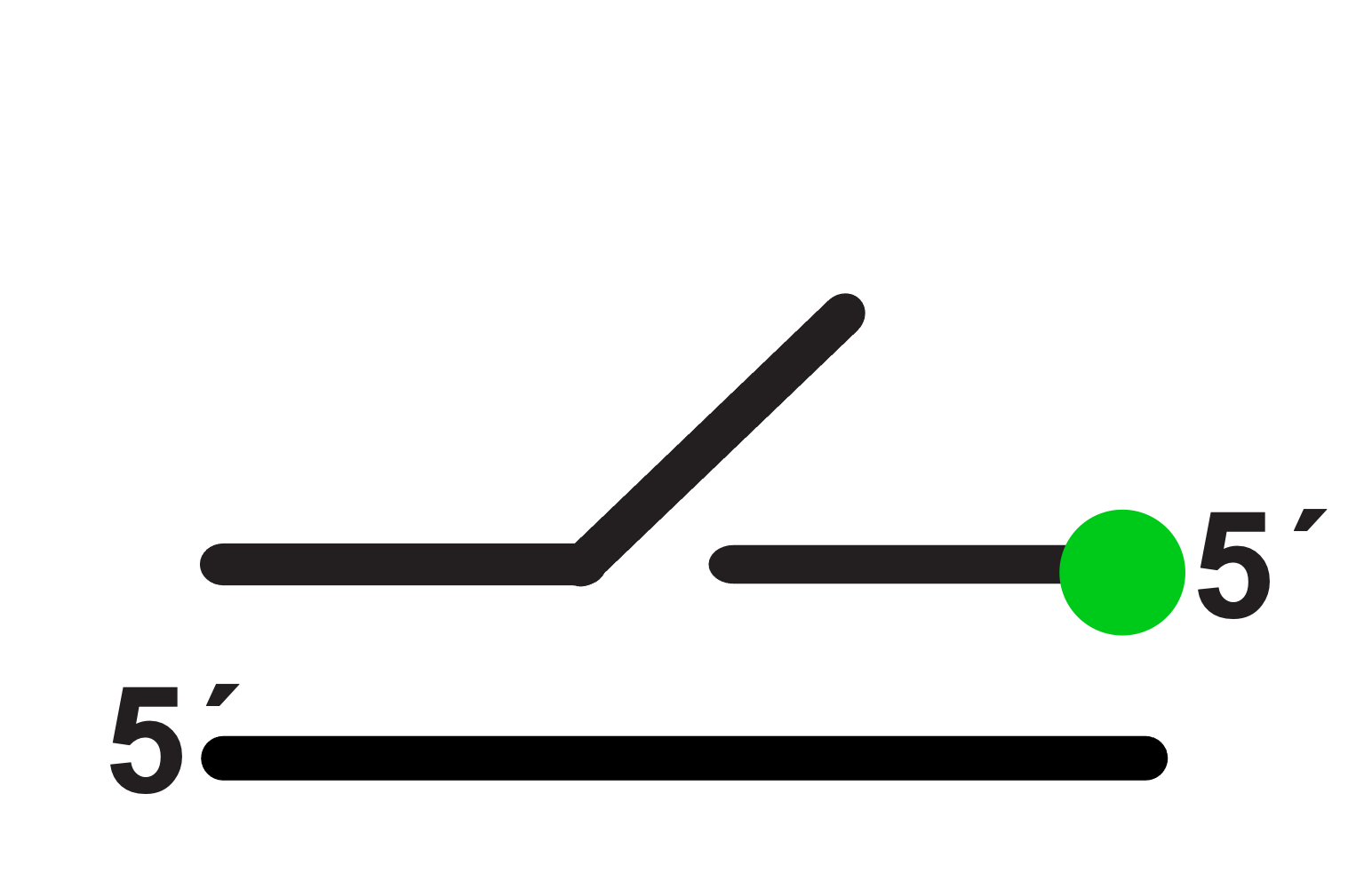 |
| **5C** | **OligoM + OligoN + OligoO** | 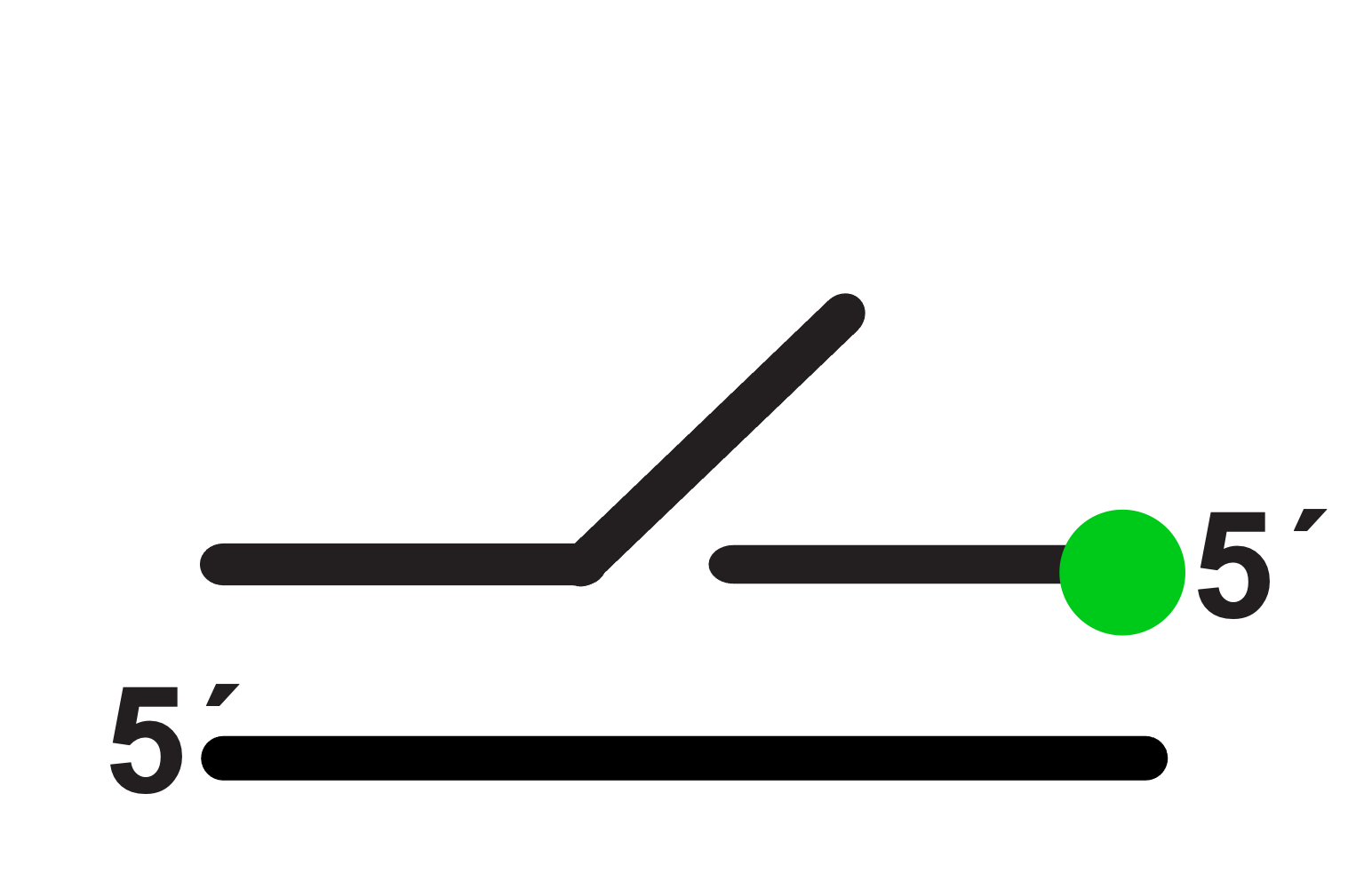 |

**Table S4. Oligonucleotides used for the analysis of plants**

| **Primer** | **Sequence** | **Aim** |
| --- | --- | --- |
| ph1 LP | 5'TTCACACTTGAATCCCAAAGAG3' | genotyping |
| ph1 RP | 5'CCAAATGCATACACATGGAAAC3' | genotyping |
| LBb1.3 | 5'ATTTTGCCGATTTCGGAAC3' | genotyping |
| ph2 LP | 5’ATTCACAACACCAAATCAGCC3’ | genotyping |
| ph2-RP | 5’CGGAGACAGAGTATGTCAGCC3’ | genotyping |
| p745 | 5’AACGTCCGCAATGTGTTAAGTTGTG3’ | genotyping |
| Fw_EMB2386 | 5’CTCTCGTTCCAGAGCTCGCAAAA3’ | RT-qPCR  Housekeeping gene |
| Rev_EMB2386 | 5’AAGAACACGCATCCTACGCATCC3´ | RT-qPCR  Housekeeping gene |
| Fw_TWINKLE | 5’GGTACTGATCAGTTTGGTGA3’ | RT-qPCR |
| Rev_TWINKLE | 5´CCTCACCAATGGTAACCCA3’ | RT-qPCR |
| Fw_UBC-21 | 5’TTC GTT CTC TTT GGG AAA TTA GA3’ | qPCR  nucleus DNA |
| Rev_UBC-21 | 5’CTC GCT GTA CCT CTT TGT ATT CTT T3’ | qPCR  nucleus DNA |
| 5'AtRpoTp | 5’GGAAGCCGTCTGCTAGAACTA 3’ | qPCR  nucleus DNA |
| 3’AtRpoTp | 5’TGTCTGAATGCAGGTCGAAAC3’ | qPCR  nucleus DNA |
| Fw_ccmFn2 | 5¨CGT GTC GTT CGT AAT GGA AA3’ | qPCR  mitochondrial DNA |
| Rev_ccmFn2 | 5’TGA TAA GCC CAC CAA CTT CC3’ | qPCR mitochondrial DNA |
| Fw_cox1 | 5´GTA GCT GCG GTG AAG TAG GC3’ | qPCR  Mitochondrial dNA |
| Rev_cox1 | 5’CTG CCT GGA TTC GGT ATC AT3’ | qPCR  Mitochondrial DNA |
| Fw_rbcL | 5’GTG TTG GGT TCA AAG CTG GT3’ | qPCR  Chloroplast DNA |
| Rev_rbcl | 5´CAT CGG TCC ACA CAG TTG TC3 | qPCR  chloroplast DNA |
| Fw_ycf2 | 5´TAG CCC TCG GTC TAT TGG TG3´ | qPCR  chloroplast DNA |
| Rev_ycf2 | 5´GGA TCC ACT TTT TGG GGA AT3’ | qPCR  chloroplast DNA |
