# Supplementary material for: A Bacteriophage‐Derived Primase‐Helicase Orchestrates Plant Organellar DNA Replication
Source: Physiol Plant. 2025 Jul 7;177(4):e70379. doi: 10.1111/ppl.70379 (PMC12230644; doi:10.1111/ppl.70379)
Supplement: Supplementary file 2 — Figure S1. Structural alignment of AtTwinkle in comparison to human Twinkle and T7 Primase‐helicase focusing on the conserved helicase motifs. Figure S2. AlphaFold structural model of AtTwinkle, AtPollA, and AtPollB. [file PPL-177-e70379-s001.docx]

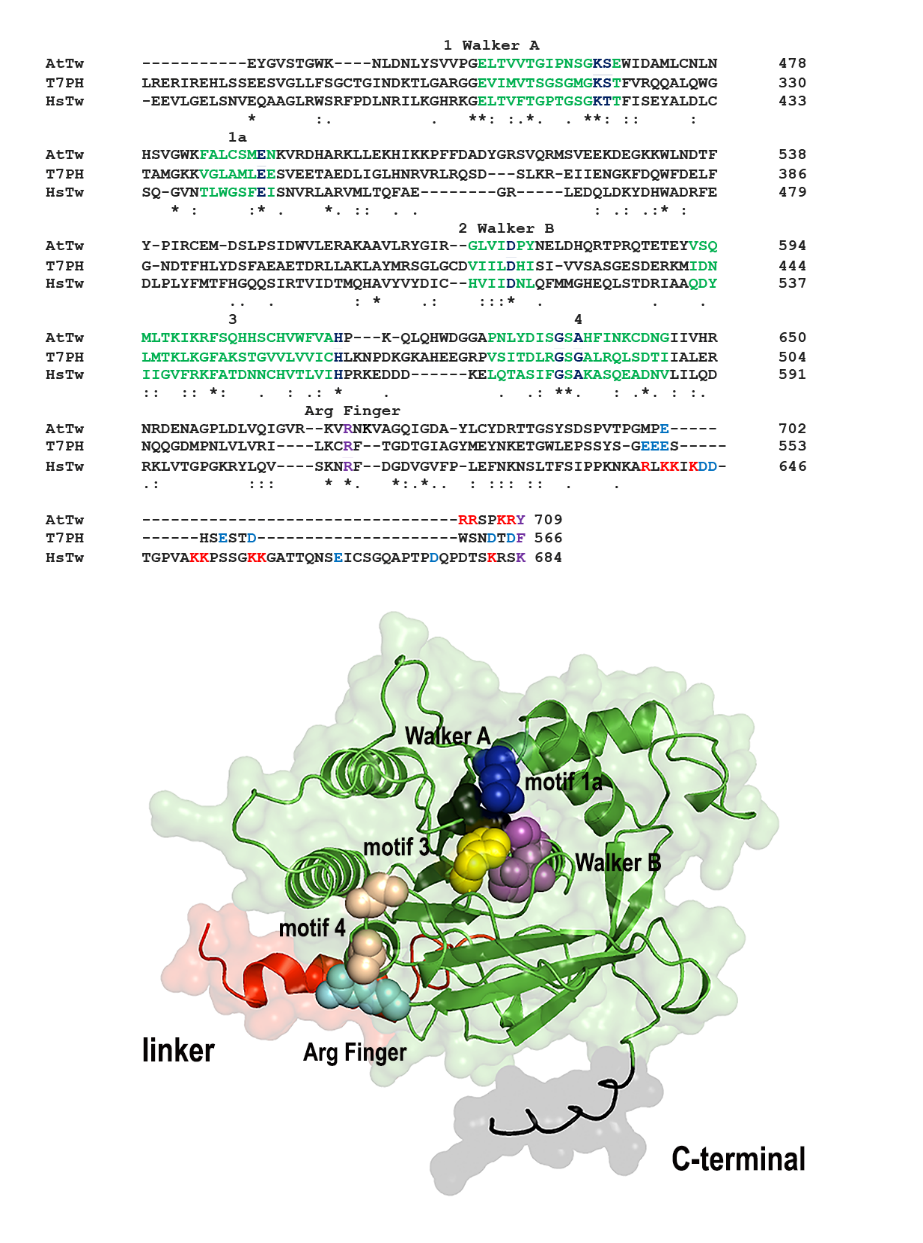


**Fig. S1 Structural alignment of AtTwinkle in comparison to human Twinkle and T7 Primase-helicase focusing on the conserved helicase motifs**. Motif 1, also dubbed Walker A, contains residues K466 and S467 that are necessary for nucleotide binding, whereas motif 1a harbors an essential glutamate (E491) indispensable for nucleotide hydrolysis (E343 in T7 helicase-primase). Other key amino acids, like residue D573 located in motif 2 (or Walker B motif) (D424 in T7 helicase-primase), residue H615 situated in motif 3 (H465 in T7 helicase-primase), residues G634 and A636 located in motif 4 (G488 and G490 in T7 helicase-primase) and the arginine finger residue R670 (R522 in T7 helicase-primase) are also present in AtTwinkle. Those residues directly participate in nucleotide hydrolysis and dsDNA unwinding (Sawaya et al. 1999, Crampton et al. 2004, Satapathy et al. 2010, Gao et al. 2019).


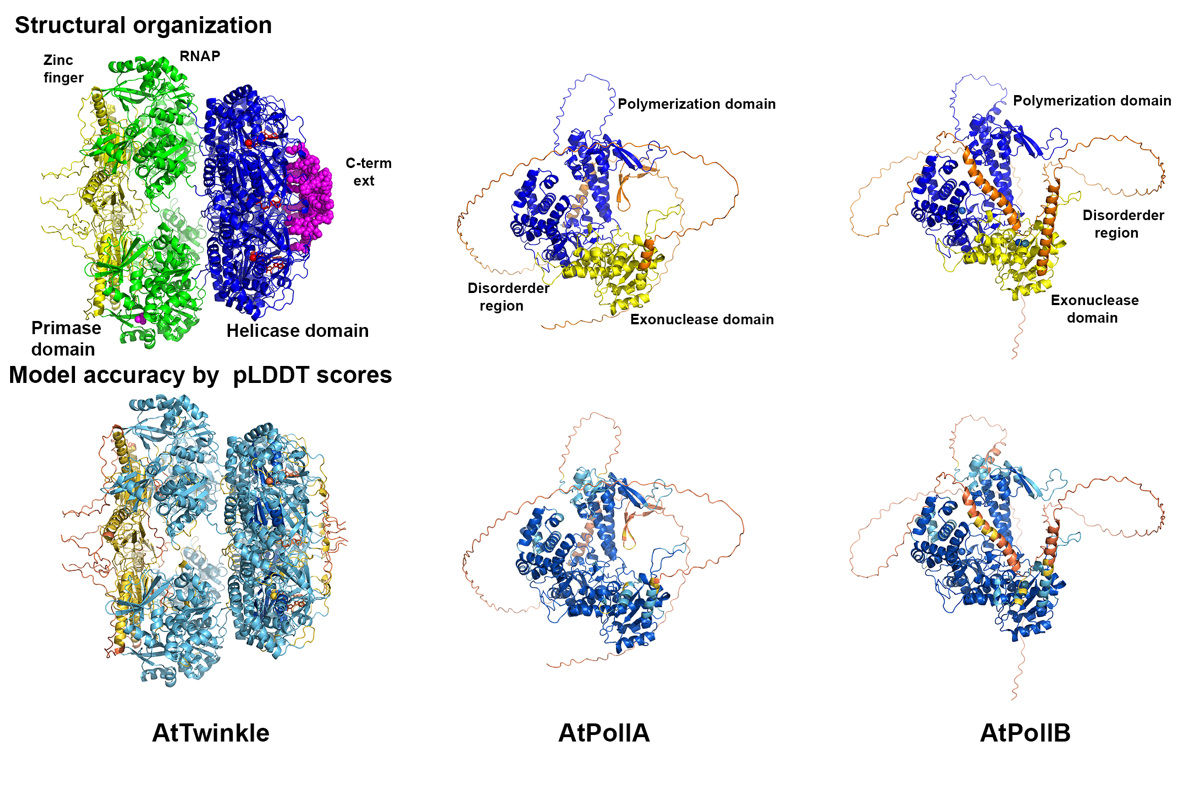


**Fig. S2 AlphaFold structural model of AtTwinkle, AtPollA, and AtPollB.** Ribbon representations of AtTwinkle, AtPoIlA, AtPoIlB, shown colored either by structural domains (upper panel) or by per-residue confidence scores (pLDDT). The pLDDT scores generated by AlphaFold, ranging from 0 to 100, are mapped to specific structural elements or domains within the mature form of the full-length proteins, following the removal of the predicted organellar transit peptides. In the color scheme used, blue corresponds to pLDDT scores >90, cyan to 70–90, yellow to 50–70, and orange to <50. Scores above 90 indicate very high prediction confidence, typically reflecting highly accurate backbone and side chain modeling. Scores between 70 and 90 represent reliable backbone predictions with potential side chain inaccuracies. Regions with pLDDT values between 50 and 70 are often associated with intrinsic disorder or structural flexibility, whereas scores below 50 indicate low confidence and poorly predicted regions. In AtPolIs, the segment spanning residues 30–257, identified as a disordered region, exhibited pLDDT values below 50. Conversely, the exonuclease and polymerase domains showed scores above 70, indicating high-confidence structural predictions, and are colored yellow and blue, respectively. In AtTwinkle, the Zinc finger subdomain (residues 118–173) presented intermediate confidence values (pLDDT 50–70), while the RNAP domain (residues 174–400) and the linker plus helicase domains (residues 401–701) displayed pLDDT values between 70 and 90, indicating confident predictions. The zinc finger is colored yellow, the RNAP domain green, and the helicase domain blue. The C-terminal 10 residues of the helicase domain are shown in purple and rendered as spheres to highlight this region.
